# Supplementary material for: Pathway selectivity in Frizzleds is achieved by conserved micro-switches defining pathway-determining, active conformations
Source: Nat Commun. 2023 Jul 29;14:4573. doi: 10.1038/s41467-023-40213-0 (PMC10387068; doi:10.1038/s41467-023-40213-0)
Supplement: Supplementary file 1 — Supplementary Information [file 41467_2023_40213_MOESM1_ESM.pdf]

Supplementary Figures S1-S18, Supplementary Tables S1-S6

Pathway selectivity in Frizzleds is achieved by conserved micro-switches defining pathway-determining, active conformations

Authors: Lukas Grätz[1,#], Maria Kowalski-Jahn[1,#], Magdalena M Scharf[1], Paweł Kozielewicz[1], Michael Jahn[2,3], Julien Bous[1], Nevin A Lambert[4], David E Gloriam[5], Gunnar Schulte[1,*]

Affiliations:

[1]Karolinska Institutet, Dept. Physiology & Pharmacology, Sec. Receptor Biology & Signaling, Biomedicum 6D, S-17165 Stockholm, Sweden.

[2]School of Engineering Sciences in Chemistry, Biotechnology and Health Science for Life Laboratory, KTH – Royal Institute of Technology, S-17121 Solna, Sweden.

[3]Max Planck Unit for the Science of Pathogens, Bioinformatics Platform, Charitéplatz 1, D-10117 Berlin, Germany.

[4]Department of Pharmacology and Toxicology, Medical College of Georgia, Augusta University, Augusta, Georgia, United States of America.

[5]Department of Drug Design and Pharmacology, University of Copenhagen, Copenhagen, Denmark.

[#] Authors contributed equally

*To whom correspondence should be addressed: gunnar.schulte@ki.se

CONTENTS

Supplementary Figure S1: Sequence alignment of the 10 FZD paralogs and SMO 3

Supplementary Figure S2: Assessment of β-catenin-dependent signaling for non-/weakly expressed FZD_5 micro-switch mutants 5

Supplementary Figure S3: Validation of FZD_5-Nluc micro-switch mutants 6

Supplementary Figure S4: Dependence of DEP recruitment to FZD_5-Nluc on receptor expression 7

Supplementary Figure S5: Characterization and validation of FZD_5-Halo-Nluc conformational sensors 8

Supplementary Figure S6: BRET over Nluc luminescence plots used for the determination of $BRET_0$. 9

Supplementary Figure S7: Combined effects of receptor mutations 10

Supplementary Figure S8: Correlation analysis of the performed assays 12

Supplementary Figure S9: Backbone RMSD plots for wt FZD_5 and micro-switch mutant MD simulations 13

Supplementary Figure S10: Per-replica plots of selected measurements 14

Supplementary Figure S11: Movements of TM5 and TM6 with a focus on $L^{5x62}A$ 16

Supplementary Figure S12: Structural rearrangements on the intracellular side of mutant $W^{7x55}A$ compared to wild-type FZD_5 18

Supplementary Figure S13: Volumetric maps for the backbone of ECL3 and ICL3. 20

Supplementary Figure S14: Variability of helix 8 flexibility between the micro-switch mutants and wild-type FZD_5. 21

Supplementary Figure S15: Interaction fingerprint heatmaps from wild-type FZD_5 and micro-switch mutant MD simulations 23

Supplementary Figure S16: Functional investigation of selected micro-switch mutants in FZD_4 25

Supplementary Figure S17: Functional investigation of selected micro-switch mutants in FZD_{10} 27

Supplementary Figure S18: Comparison of parameters from DEP titration experiments with FZD paralogs ... 29

Supplementary Table S1: Primers used in the study 30

Supplementary Table S2: p values for indicated FZD_5 micro-switch mutants from the different ELISA assays 34

Supplementary Table S3: log $BRET_{50}$ and $BRET_{max}$ values from DEP-Venus titration experiments 35

Supplementary Table S4: % BRET values from experiments with G_q 4A 36

Supplementary Table S5: $BRET_0$ values from experiments with the FZD_5-Halo-Nluc conformational sensor ... 37

Supplementary Table S6: Templates used for the modeling of the FZD_5 structure 38

References 38

Supplementary Figure S1

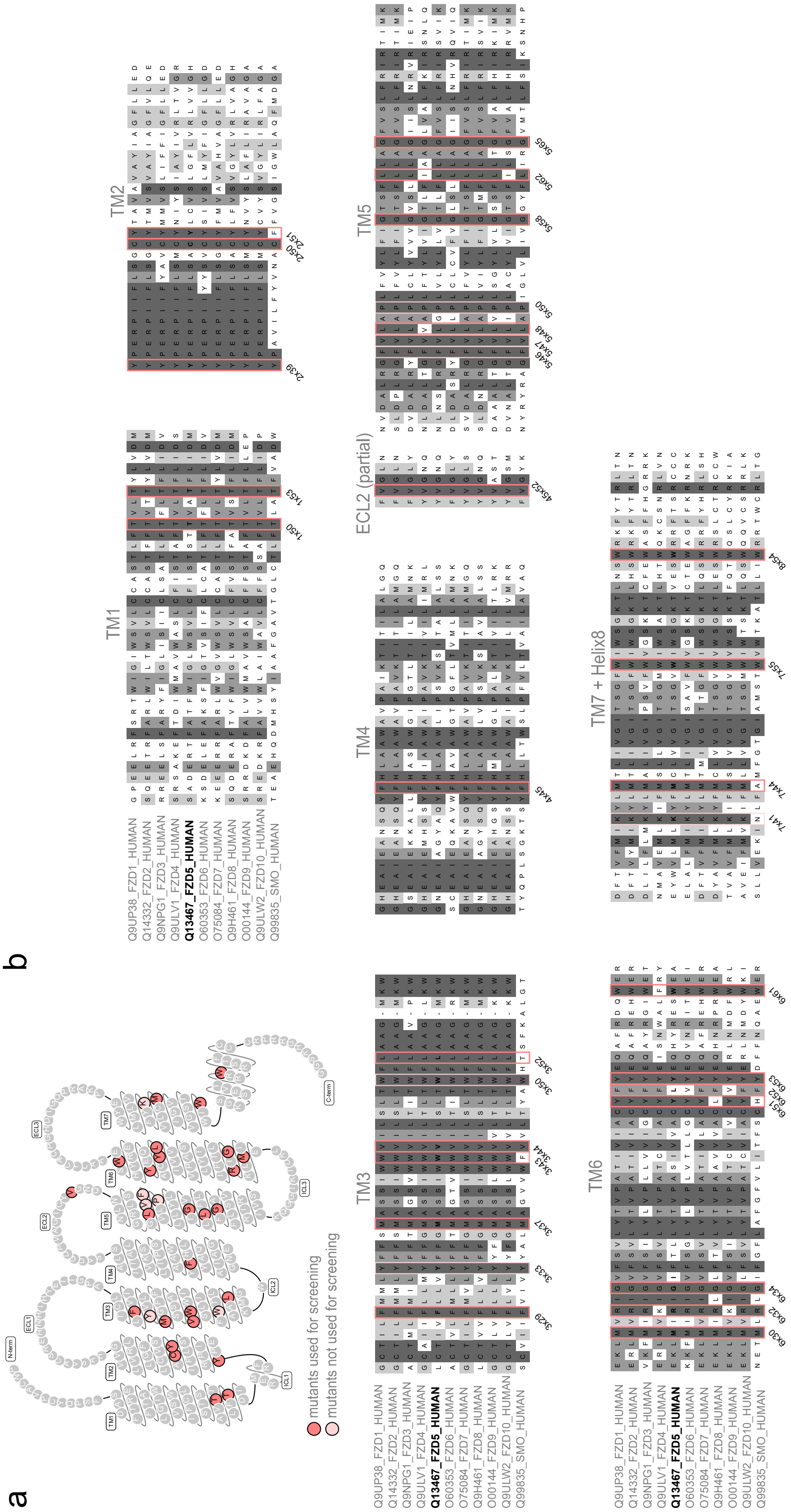

Supplementary Fig. S1: Sequence alignment of the 10 FZD paralogs and SMO. (**a**) Snake plot of human FZD_5 with mutated amino acid residues highlighted in light red (amino acids not used for screening) and red (amino acids used for screening). The N and C termini of FZD_5 were omitted for clarity. (**b**) Class F sequence alignment visualizing representative parts of the receptor with the relevant micro-switch mutants. The different shades of gray illustrate sequence conservation ranging from white (not conserved) to dark gray (conserved). C-term, C terminus; ECL, extracellular loop; ICL, intracellular loop; N-term, N terminus.

Supplementary Figure S2

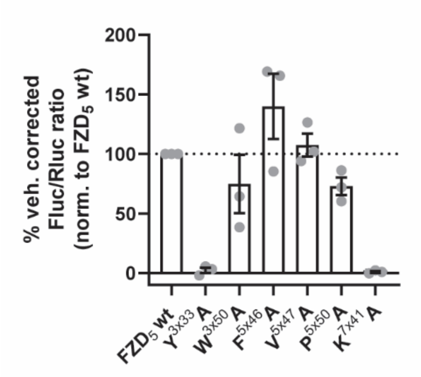

Supplementary Fig. S2: Assessment of β-catenin-dependent signaling for non-/weakly expressed FZD_5 micro-switch mutants. TOPFlash reporter gene responses were measured in $\Delta FZD_{1\text{-}10}$ HEK293T cells, transiently transfected with indicated FZD_5 micro-switch mutants or wild-type FZD_5, after stimulation with recombinant WNT-3A (300 ng/mL). Data show mean ± SEM of three independent experiments performed in duplicate and represent vehicle-corrected Fluc/Rluc ratios normalized to wild-type FZD_5 (included in every experiment).

Supplementary Figure S3

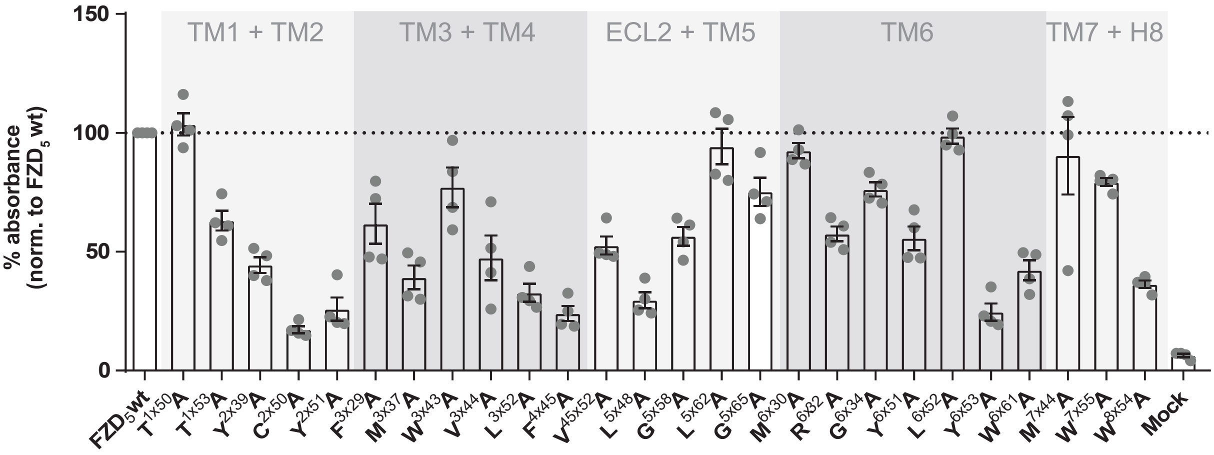

Supplementary Fig. S3: Validation of FZD_5-Nluc micro-switch mutants. Cell surface expression in HEK293A cells, transiently transfected with the different FZD_5-Nluc micro-switch mutants, wild-type FZD_5-Nluc, or pcDNA3.1 (Mock), was quantified by whole-cell ELISA using an antibody directed against the N-terminal HA tag. Data show mean ± SEM of four independent experiments performed in triplicate and mean values were normalized to wild-type FZD_5-Nluc surface expression. Statistical differences between the FZD_5-Nluc constructs and Mock were assessed using one-way ANOVA (matched, with Geisser-Greenhouse correction), followed by an uncorrected Fisher's LSD post-hoc test. Note that values for all micro-switch mutants are significantly different from Mock (pcDNA3.1 control). Corresponding significance levels are shown in **Supplementary Table S2**.

Supplementary Figure S4

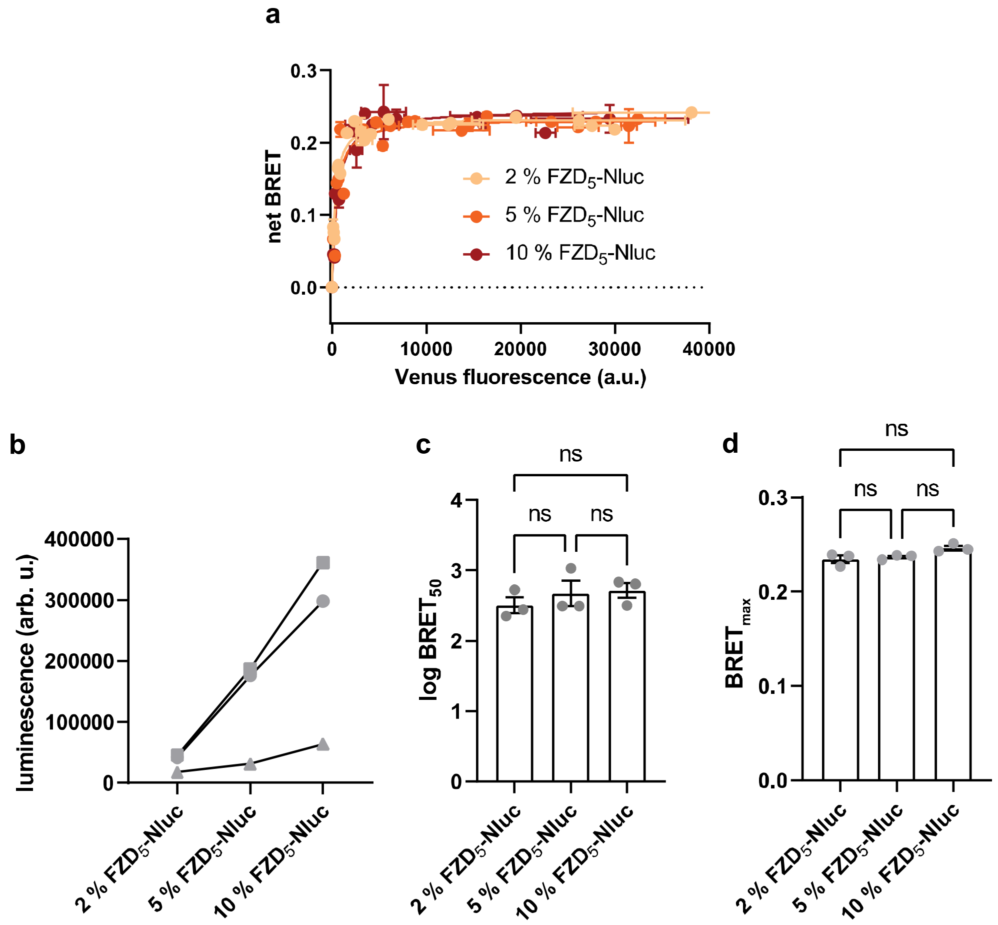

Supplementary Fig. S4: Dependence of DEP recruitment to FZD_5-Nluc on receptor expression. (**a**) Recruitment of DEP-Venus to FZD_5-Nluc at different expression levels. Experiments were performed in HEK293A cells, transiently transfected with a constant plasmid amount of FZD_5-Nluc (indicated percentage refers to the total transfected DNA amount; note that 2% FZD_5-Nluc were used for experiments in **Fig. 4** in the main text) and increasing amounts of plasmid encoding DEP-Venus. Data show mean ± SD of three independent experiments (data points from different experiments are superimposed) performed in duplicate. (**b**) Luminescence values from titration experiments (no acceptor conditions) depicted in (**a**). Luminescence values from the same experiment are depicted as the same symbol (squares, circles or triangles) and are connected. (**c**, **d**) log $BRET_{50}$ (**c**) and $BRET_{max}$ (**d**) values from DEP titration experiments performed with different plasmid amounts of FZD_5-Nluc. Data show mean ± SEM of three independent experiments performed in duplicate. Statistical differences were tested using one-way ANOVA followed by Tukey's post-hoc analysis. ns: not significant. Abbreviations: arb. u.: arbitrary units.

Supplementary Figure S5

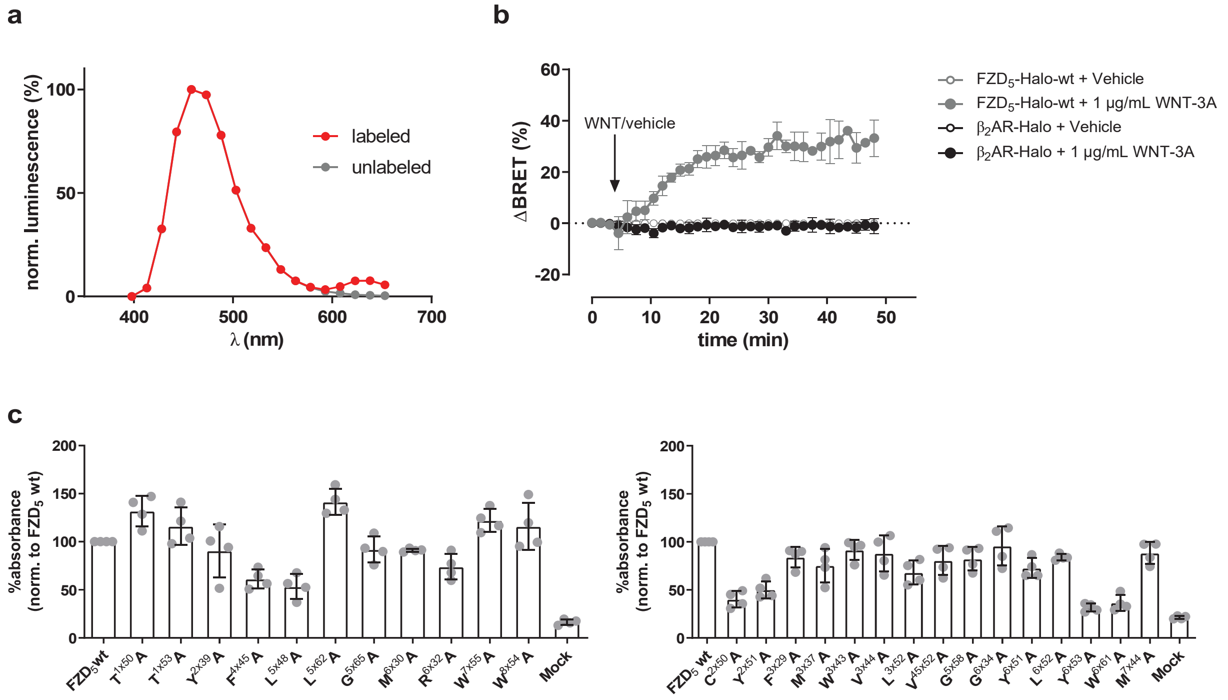

Supplementary Fig. S5: Characterization and validation of FZD5-Halo-Nluc conformational sensors. (**a**) Luminescence emission spectrum of HaloTag 618 ligand-labeled and -unlabeled FZD5-Halo-Nluc, transiently expressed in HEK293A cells. Data show mean ± SEM of five independent experiments performed in triplicate. Data were normalized to the respective donor emission peak for each experiment. (**b**) BRET response elicited by 1 µg/mL of recombinant WNT-3A in HEK293A cells, transiently transfected with wild-type FZD5-Halo-Nluc or β2AR-Halo-NLuc as a negative control. Data show mean ± SEM of three independent experiments performed in triplicate. (**c**) Surface expression analysis of the FZD5-Halo-Nluc constructs by whole-cell ELISA using an antibody directed against the N-terminal HA tag. Experiments were conducted in HEK293A cells, transiently transfected with the different FZD5-Halo-Nluc micro-switch mutants, wild-type FZD5-Halo-Nluc or pcDNA3.1 (Mock). Data show mean ± SEM of four independent experiments performed in triplicate and mean values were normalized to wild-type FZD5-Halo-Nluc (for each experiment). Statistical differences between the FZD5-Halo-Nluc constructs and pcDNA3.1 transfection were assessed using one-way ANOVA (matched, with Geisser-Greenhouse correction), followed by an uncorrected Fisher's LSD post-hoc test. Note that the values for wild-type FZD5-Halo-Nluc and all FZD5-Halo-Nluc micro-switch mutants are significantly different from pcDNA3.1 control. Corresponding significance levels are shown in **Supplementary Table S2**.

Supplementary Figure S6

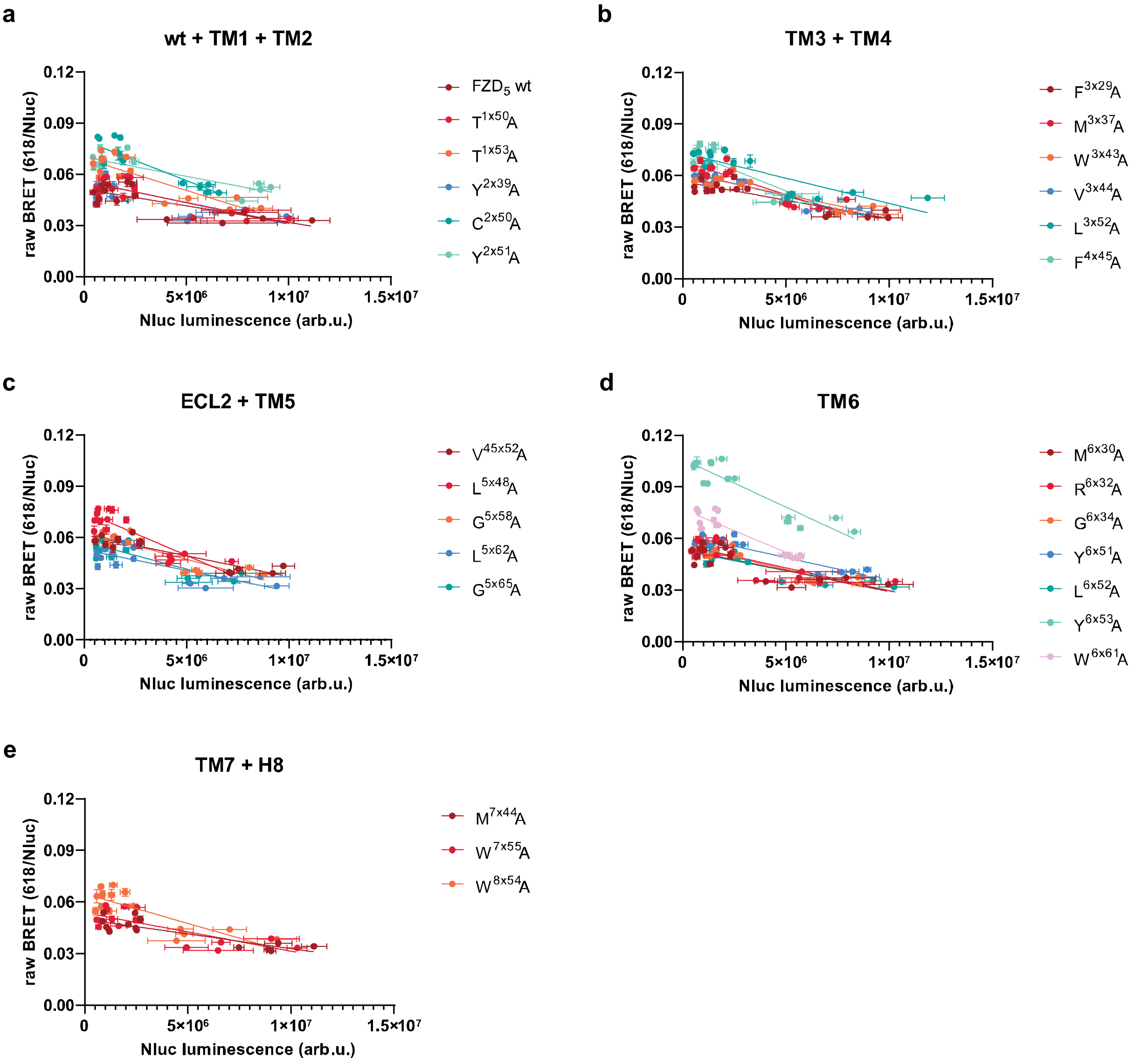

Supplementary Fig. S6: BRET over Nluc luminescence plots used for the determination of BRET$_0$. Datapoints were obtained from experiments performed in HEK293A cells, transiently transfected with wild-type FZD$_5$-Halo-Nluc (**a**) or the indicated FZD$_5$-Halo-Nluc micro-switch mutants located in TMs 1 and 2 (**a**), TMs 3 and 4 (**b**), ECL2 and TM5 (**c**), TM6 (**d**) or TM7 and H8 (**e**). Data show mean ± SD of five independent experiments performed in sextuplicate (three different timepoints after substrate addition per experiment) and were fitted to a linear correlation. Numerical values for BRET$_0$, R^2 values of the linear fits and p-values for the runs test for deviation from a linear correlation are listed in **Supplementary Table S5**. Abbreviations: arb. u.: arbitrary units.

Supplementary Figure S7

Mutant	Surf expr	DVL shift	TOP Flash	DEP recruitment log $BRET_{50}$	DEP recruitment BRET max	G_q 4A	Conf sensor	No. effects
$T^{1x50}A$		■	■	■			■	4
$T^{1x53}A$	■	■	■	■	■	■	■	7
$Y^{2x39}A$		■	■	■	■		■	5
$C^{2x50}A$	■				■	■	■	4
$Y^{2x51}A$	■				■	■	■	4
$F^{3x29}A$	■				■		■	3
$Y^{3x33}A$	■	nd	nd	nd	nd	nd	nd	nd
$M^{3x37}A$	■			■	■	■	■	5
$W^{3x43}A$							■	1
$V^{3x44}A$	■		■				■	3
$W^{3x50}A$	■	nd	nd	nd	nd	nd	nd	nd
$L^{3x52}A$	■		■		■	■	■	5
$V^{45x52}A$	■		■		■	■	■	5
$F^{4x45}A$	■	■		■	■	■	■	6
$F^{5x46}A$	■	nd	nd	nd	nd	nd	nd	nd
$V^{5x47}A$	■	nd	nd	nd	nd	nd	nd	nd
$L^{5x48}A$	■				■	■	■	4
$P^{5x50}A$	■	nd	nd	nd	nd	nd	nd	nd
$G^{5x58}A$	■					■	■	3
$L^{5x62}A$		■	■					2
$G^{5x65}A$	■	■				■	■	4
$M^{6x30}A$			■	■				2
$R^{6x32}A$	■	■	■	■	■	■	■	7
$G^{6x34}A$		■	■				■	3
$Y^{6x51}A$	■					■	■	3
$L^{6x52}A$		■	■					2
$Y^{6x53}A$	■		■	■	■	■	■	6
$W^{6x61}A$	■		■		■	■	■	5
$K^{7x41}A$	■	nd	nd	nd	nd	nd	nd	nd
$M^{7x44}A$		■	■					2
$W^{7x55}A$		■	■	■	■			4
$W^{8x54}A$		■	■		■	■	■	5
Effect:	22	12	16	9	15	15	21	Avg: 4
Effect (%):	69	46	62	35	58	58	81	

Supplementary Fig. S7. Combined effects of receptor mutations. Heatmap of mutation effects across all experimentally evaluated functional parameters. Six mutants were not expressed at the cell surface and were therefore non-determined (marked “nd” in the heatmap) in the subsequent functional profiling. These non-expressing mutants are excluded in the shown average number of effects per mutant (4,0) and percent of mutants with effect (35-81). The cut-offs (semi-quantitative) used for the effects were: surface expression (20% reduction, only HA-FZD_5-1D4 backbone), DVL shift (+/- 50%), TOPFlash (40% reduction), DEP recruitment log $BRET_{50}$ (+/- 0.4), DEP recruitment $BRET_{max}$ (+/- 0.03), G_q 4A coupling (+/- 4%) and conformational sensor (+/- 0.003) – all as compared to the wild-type FZD_5. The raw values are available in **Supplementary Data 2.**

Supplementary Figure S8

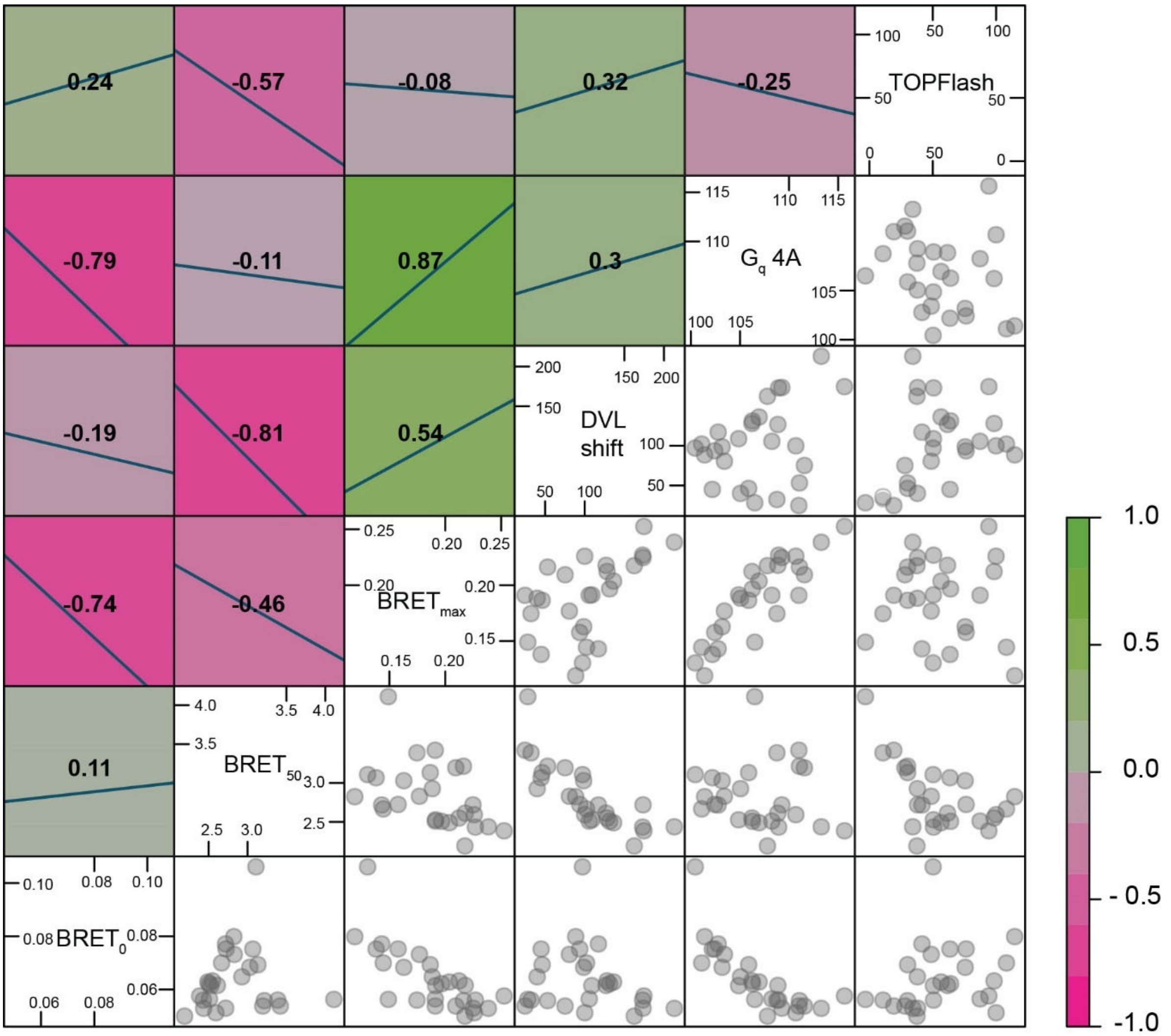

Supplementary Fig. S8: Correlation analysis of the performed assays. Scatter plots showing pairwise correlation of all variables against each other. Lower triangle shows arithmetic mean values for each mutant as dots. Upper triangle shows the coefficient of correlation for each pair of variables shown in the lower triangle (number). The color encodes strength of correlation between -1.0 (pink) and 1.0 (green). Lines represent the fits of a linear regression.

Supplementary Figure S9

a: wild-type FZD$_5$

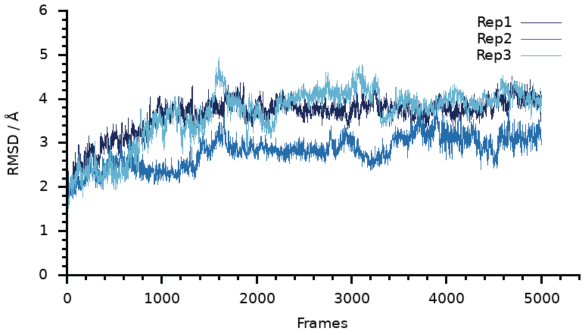

b: Y^{6x53}A

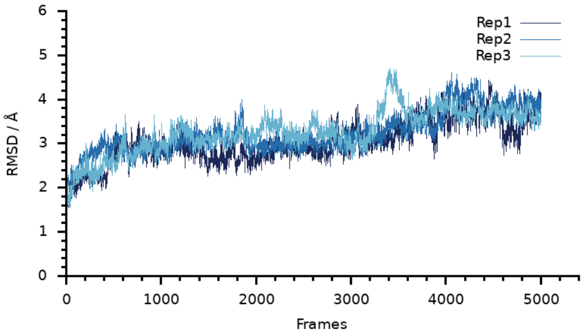

c: C^{2x50}A

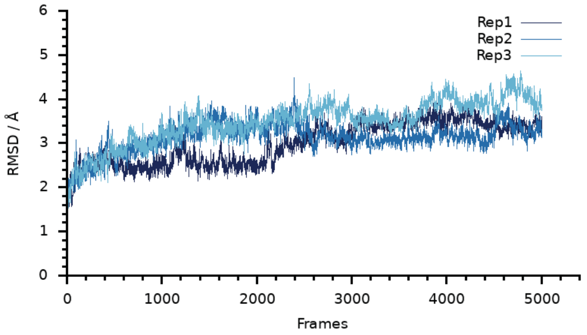

d: Y^{2x51}A

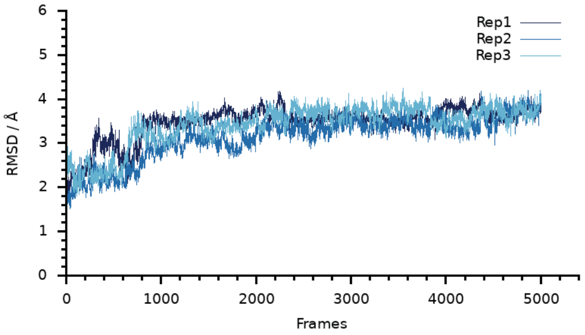

e: L^{5x62}A

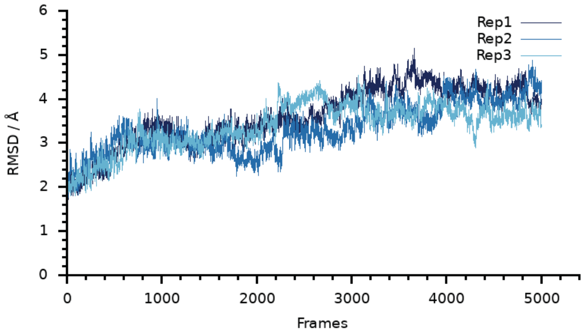

f: G^{5x65}A

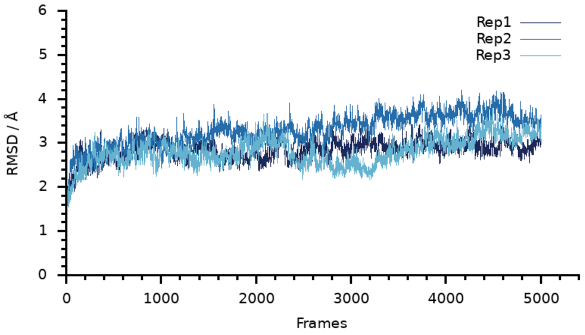

h: R^{6x32}A

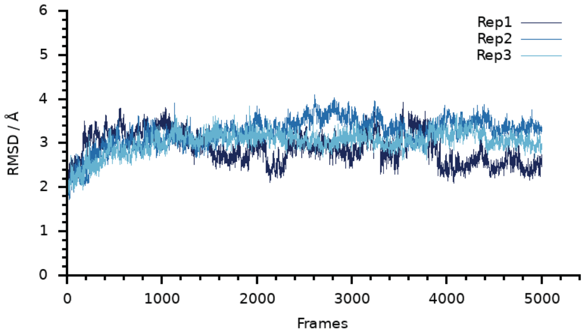

i: W^{7x55}A

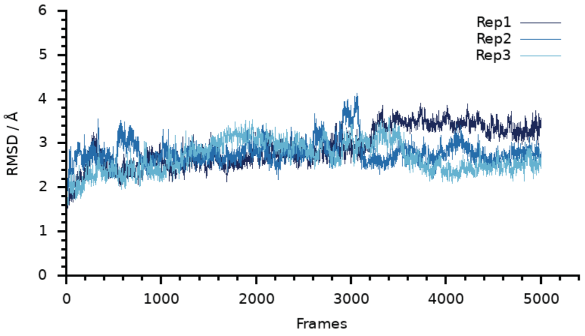

Supplementary Fig. S9: Backbone RMSD plots for wild-type FZD$_5$ and micro-switch mutant MD simulations. The RMSD of all Cα atoms was calculated in reference to the initial FZD$_5$ model to allow for better comparability. RMSD is plotted over the entire trajectory of 500 ns or 5000 frames and each of the three independent replicas.

Supplementary Figure S10

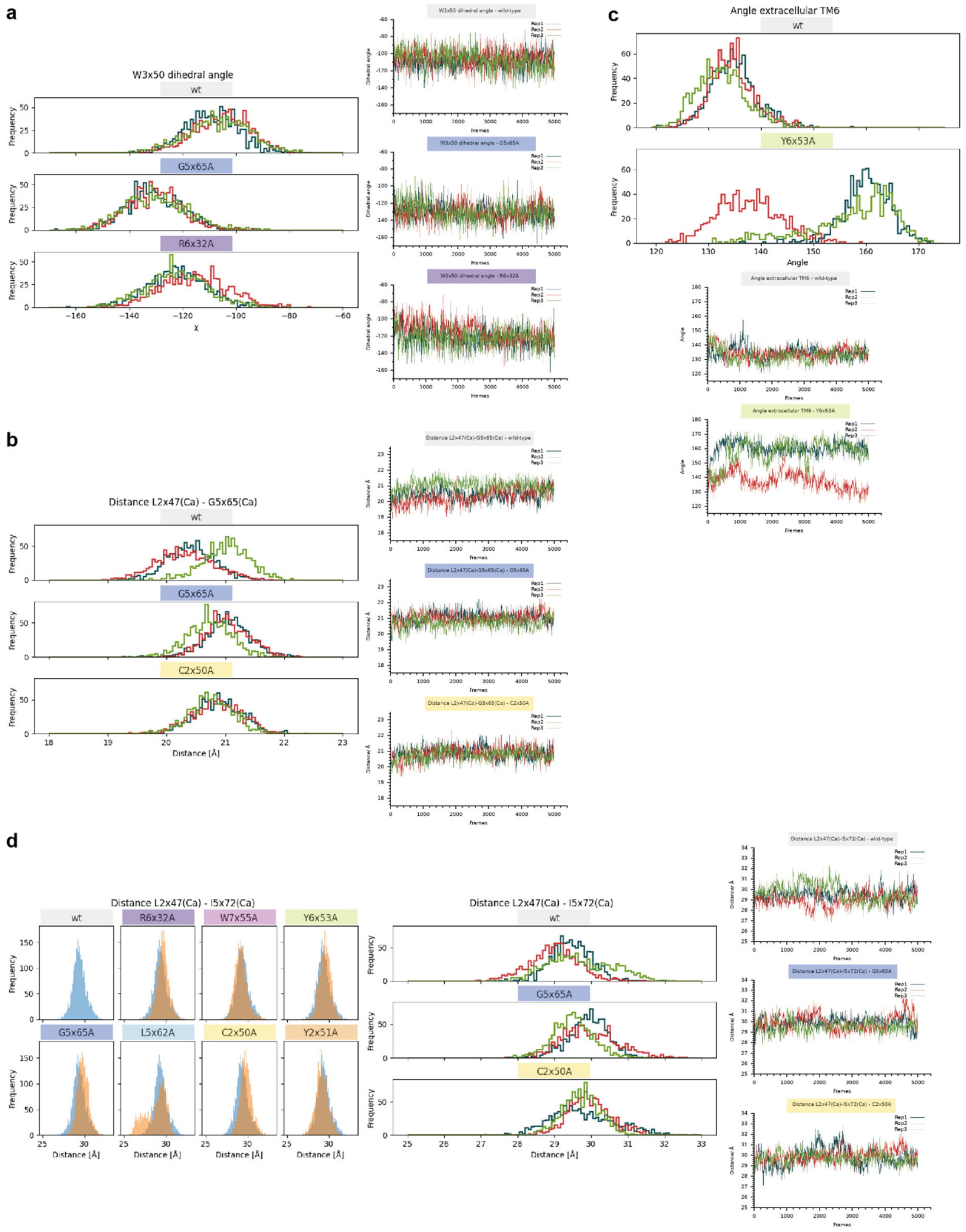

Supplementary Fig. S10: Per-replica plots of selected measurements. (**a**)-(**c**) Per-replica histograms and traces over time for selected mutants compared to wild-type FZD_5 for measurements discussed in **Fig. 8**. Data points were measured for every 5th frame in all plots. (**a**) Dihedral angle of residue W^{3x50} for the individual replica of wild-type FZD_5, $G^{5x65}A$ and $R^{6x32}A$, plotted as histograms and traces over 5000 frames (500 ns), corresponding to **Fig. 8c**. The observed change of the dihedral angle of W^{3x50} for $G^{5x65}A$ and to a lesser extent $R^{6x32}A$ compared to wild-type FZD_5 is reproducible over all three replicas. (**b**) Distance between Cα atoms of residues L^{2x47} and G^{5x65} for the individual replicas of wild-type FZD_5, $G^{5x65}A$ and $C^{2x50}A$ plotted as histograms and trace over 5000 frames (500 ns), corresponding to **Fig. 8d**. While distance distribution and traces over time are reproducible for the mutants, wild-type FZD_5 also shows a shift towards a larger distance for one of three replicas. This argues for a variability of this distance also in wild-type FZD_5, but with a more stabilized larger distance in mutants $G^{5x65}A$ and $C^{2x50}A$. (**c**) Angle of the extracellular portion of TM6 for the individual replicas of wild-type FZD_5 and $Y^{6x53}A$ plotted as histograms and traces over 5000 frames (500 ns), corresponding to **Fig. 8f**. While the angle is reproducible for the three replicas for wild-type FZD_5, one replica of $Y^{6x53}A$ displays a smaller angle of the helix kink compared to the other two replicas. Observing the angle over time, however, shows that the TM6 angle in this replica is quite unstable compared to wild-type FZD_5 as well as the other two replicas. (**d**) Additional analysis of the distance between residues L^{2x47} and I^{5x72} at a more intracellular position of TM2 and TM5 compared to **Fig. 8d** and (**b**). The larger distance for $G^{5x65}A$ and $C^{2x50}A$ compared to wild-type FZD_5 is reproducible over three replicas and the outward shift of TM5 also for $C^{2x50}A$ is more evident for this distance.

Supplementary Figure S11

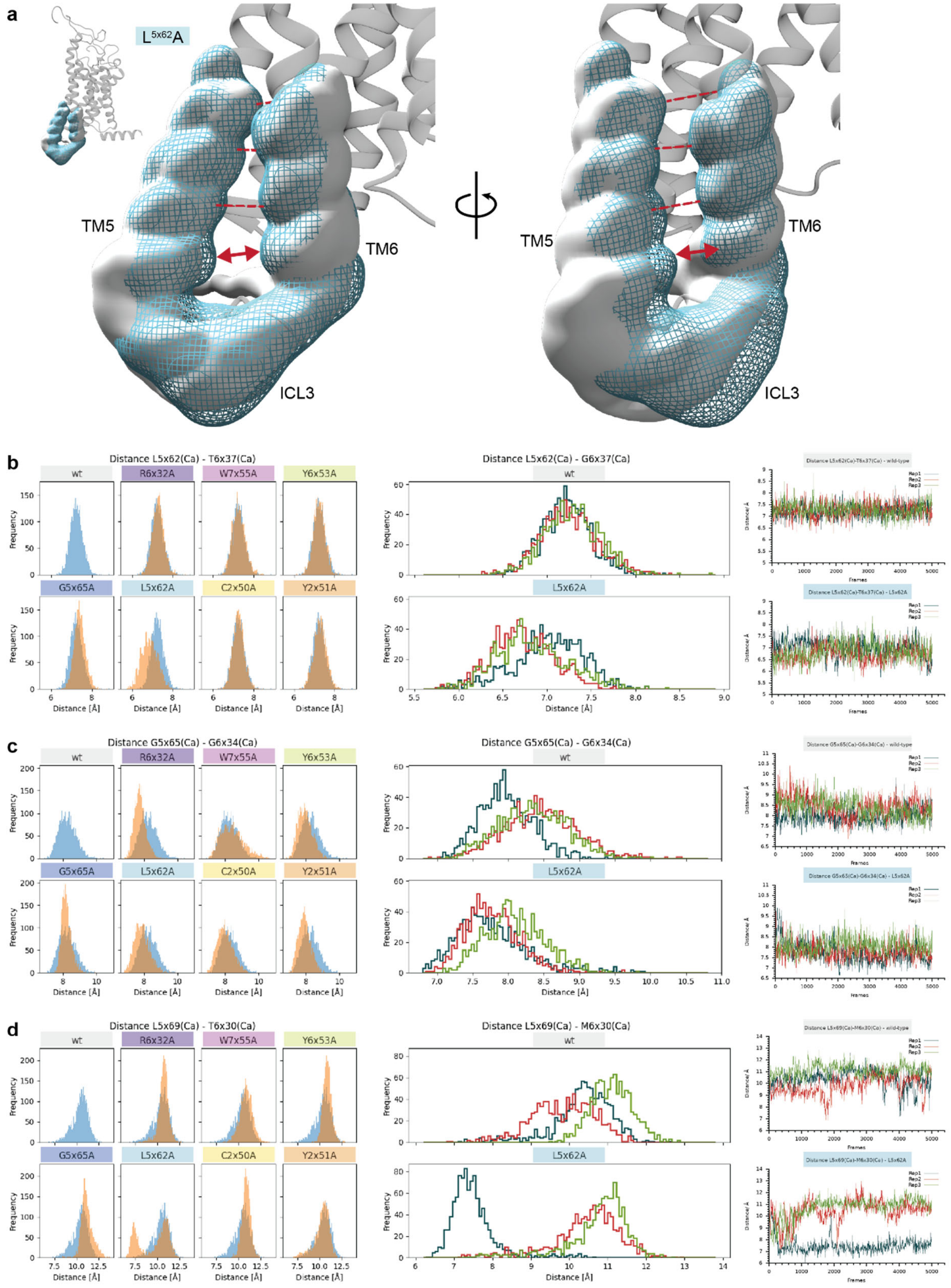

Supplementary Fig. S11: Movements of TM5 and TM6 with a focus on L^{5x62}A. (**a**) View of the volumetric maps of the receptor backbone atoms of ICL3 and the intracellular parts of TM5 and TM6 calculated over all three replicas for wild-type FZD_5 (light grey) and L^{5x62}A (cyan mesh) from two different angles. A cartoon representation of the initial FZD_5 model is aligned with the volumetric maps for clarity. The movement of TM5 and TM6 towards each other is indicated by a red arrow. The dashed lines indicate the distances measured in (**b**) (top), (**c**) (middle) and (**d**) bottom. (**b**)-(**d**) Distance between TM5 and TM6 measured between $C\alpha$ atoms of the indicated residues. Left: Histograms of occurring distances (calculated for every 5th frame of the concatenated trajectory of all three replicas) shown in blue (wild-type FZD_5) and orange (indicated mutant). Per replica histograms and traces over 5000 frames (500 ns) for every 5th frame for wild-type FZD_5 and L^{5x62}A are shown in the middle and right images. In general, the measured values are less stable, the further towards the intracellular side the distances are measured. Overall, a tendency towards smaller distances can be observed for L^{5x62}A compared to wild-type FZD_5, although with varying reproducibility over the replicas. However, this argues for a stabilization of a smaller distance between TM5 and TM6 for L^{5x62}A compared to wild-type FZD_5.

Supplementary Figure S12

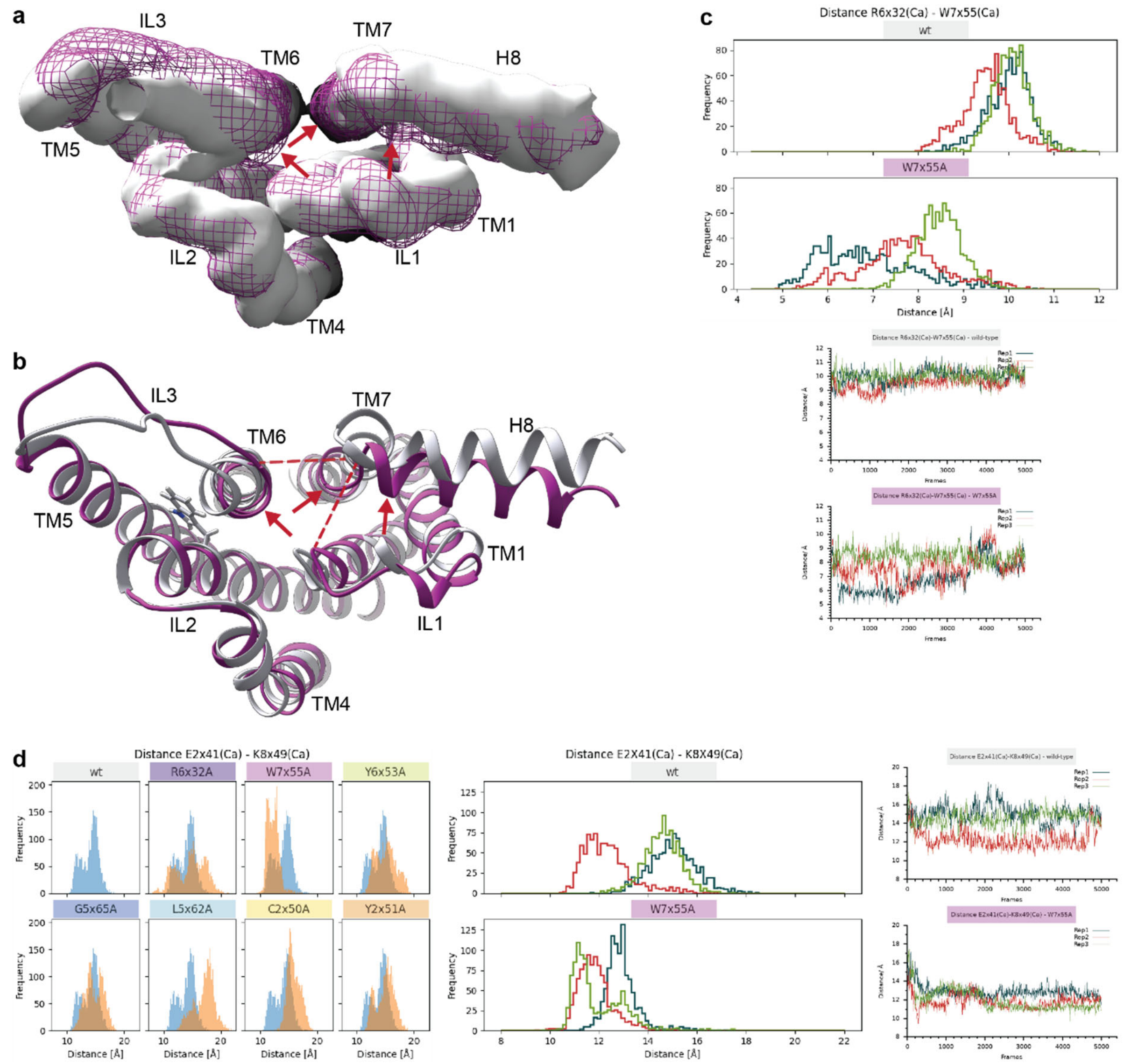

Supplementary Fig. S12: Structural rearrangements on the intracellular side of mutant W^{7x55}A compared to wild-type FZD$_5$. (**a**) Volumetric maps of the backbone movement of wild-type FZD$_5$ (light grey) and the micro-switch mutant W^{7x55}A (dark magenta mesh) as viewed from the intracellular side of the receptor. The inward movements of TM6, TM7 and H8 towards the receptor core are indicated by red arrows. (**b**) Cartoon representation of the representative structure of the main cluster (clustered on TM region over all three replicas using every 5th frame) for wild-type FZD$_5$ (light grey) and W^{7x55}A (dark magenta) viewed from the intracellular side. The inward movements of TM6, TM7 and H8 towards the receptor core are indicated by red arrows. Dashed lines indicate the distances measured between TM6 and TM7 (**Fig. 8g** and (**c**)) and between TM2 and H8 (**d**). (**c**) Distance distribution and variation over time for the individual replica of wild-type FZD$_5$ and W^{7x55}A for the distance between TM6 and TM7

corresponding to **Fig. 8g**. Although this distance varies for the different replicas of $W^{7x55}A$ it seems to converge to a similar distance for all replicas and is constantly smaller compared to wild-type FZD_5. (**d**) Histogram of the distances between the Cα atoms of E^{2x41} and K^{8x49} as measured for every 5th frame throughout the entire trajectory for wild-type FZD_5 (blue) and the indicated mutant (orange) (left). The comparatively smaller distances observed for $W^{7x55}A$ confirm the observations evident in (**a**) and (**b**). Per replica histograms of this distance (middle) and the distance over 5000 frames (500 ns; right) are shown for wild-type FZD_5 and $W^{7x55}A$. While the distance seems reproducible over all three replicas for $W^{7x55}A$, one replica of wild-type FZD_5 displays an overall smaller distance compared to the other two replica (middle). However, the smaller distance between TM2 and H8 seems to be much more stable over time for $W^{7x55}A$ compared to any of the wild-type replica, arguing for a significant conformational and dynamic change in this region for $W^{7x55}A$ compared to wild-type FZD_5.

Supplementary Figure S13

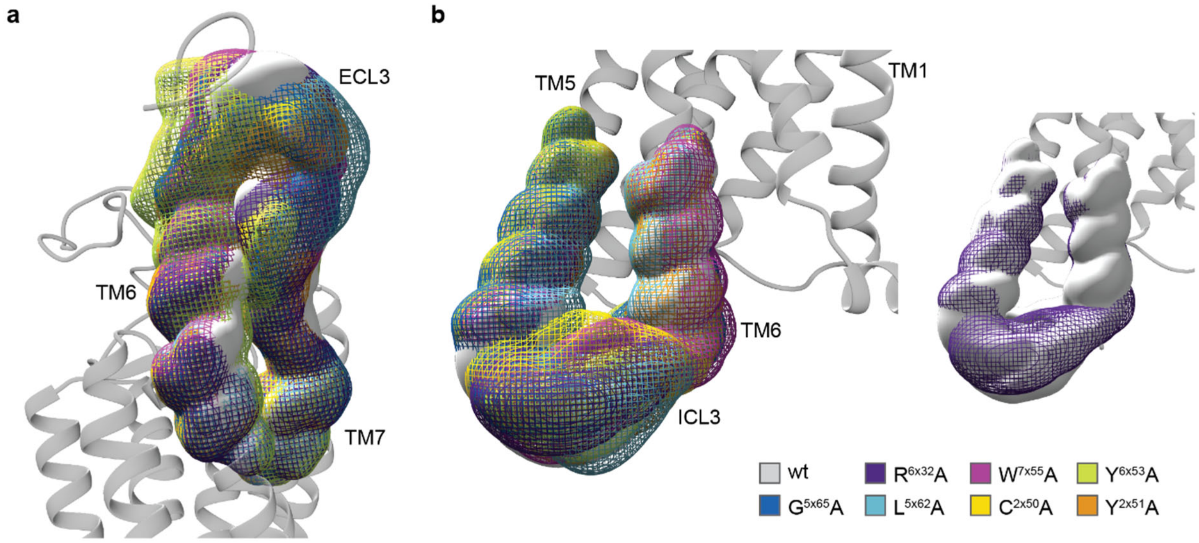

Supplementary Fig. S13: Volumetric maps for the backbone of ECL3 and ICL3. Overlay of the volumetric maps of the backbone movement over the entire trajectory of wild-type FZD_5 (light grey) and the simulated micro-switch mutants (mesh; see legend for colors). A cartoon representation of the initial FZD_5 model is aligned with the volumetric maps for clarity. (**a**) Orientation of ECL3 including the extracellular portion of TM6 and TM7. (**b**) Orientation of ICL3 including the intracellular portion of TM5 and TM6. The small inset shows $R^{6x32}A$ in comparison to wild-type FZD_5.

Supplementary Figure S14

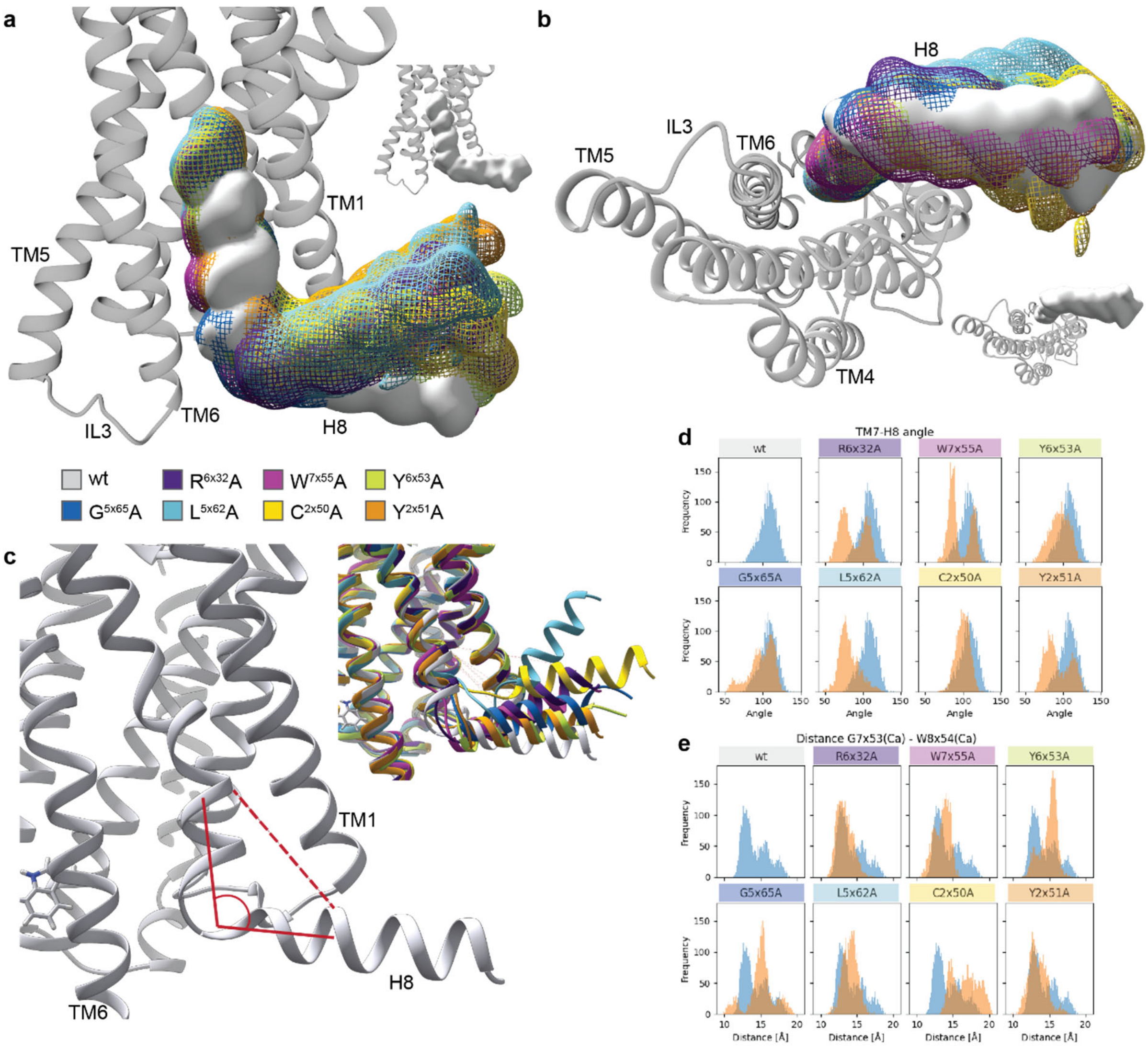

Supplementary Fig. S14: Variability of helix 8 flexibility between the micro-switch mutants and wild-type FZD_5. (**a**) and (**b**) Overlay of the volumetric maps of the backbone movement over the entire trajectory of wild-type FZD_5 (light grey) and the simulated micro-switch mutants (displayed as mesh; see legend for colors). A cartoon representation of the initial FZD_5 model is aligned with the volumetric maps for clarity. (**a**) H8 orientation from the side view and (**b**) H8 orientation as seen from the intracellular side. The small insets show the volumetric map for wild-type FZD_5 only using the same point of view. (**c**) Cartoon representation of the representative structure of the main cluster (clustered on TM region over all three replicas using every 5th frame) for wild-type FZD_5. The shown angle (in red) indicates the angle measured in (**d**) and the dashed red line the distance measured in (**e**). The smaller inset shows the overlay of the main cluster representative of wild-type (light grey) and the mutants (see legend for colors). (**d**) Histogram of the angle between TM7 and H8 for wild-type FZD_5 (blue) and the indicated

mutant (orange) as measured for every 5th frame over all three replicas. Especially for L^{5x62}A, but in parts also for some of the other mutants, a smaller angle (corresponding to a movement of the C-terminal end of H8 into the membrane) can be observed. (**e**) Histogram of the distance between TM7 and H8 for wild-type FZD_5 (blue) and the indicated mutant (orange) as measured for every 5th frame over all three replicas. Especially for C^{2x50}A an increase of the distance can be observed which (in combination with the unchanged angle in (**d**)) corresponds to a movement of H8 parallel to the membrane and away from the receptor core.

Supplementary Figure S15

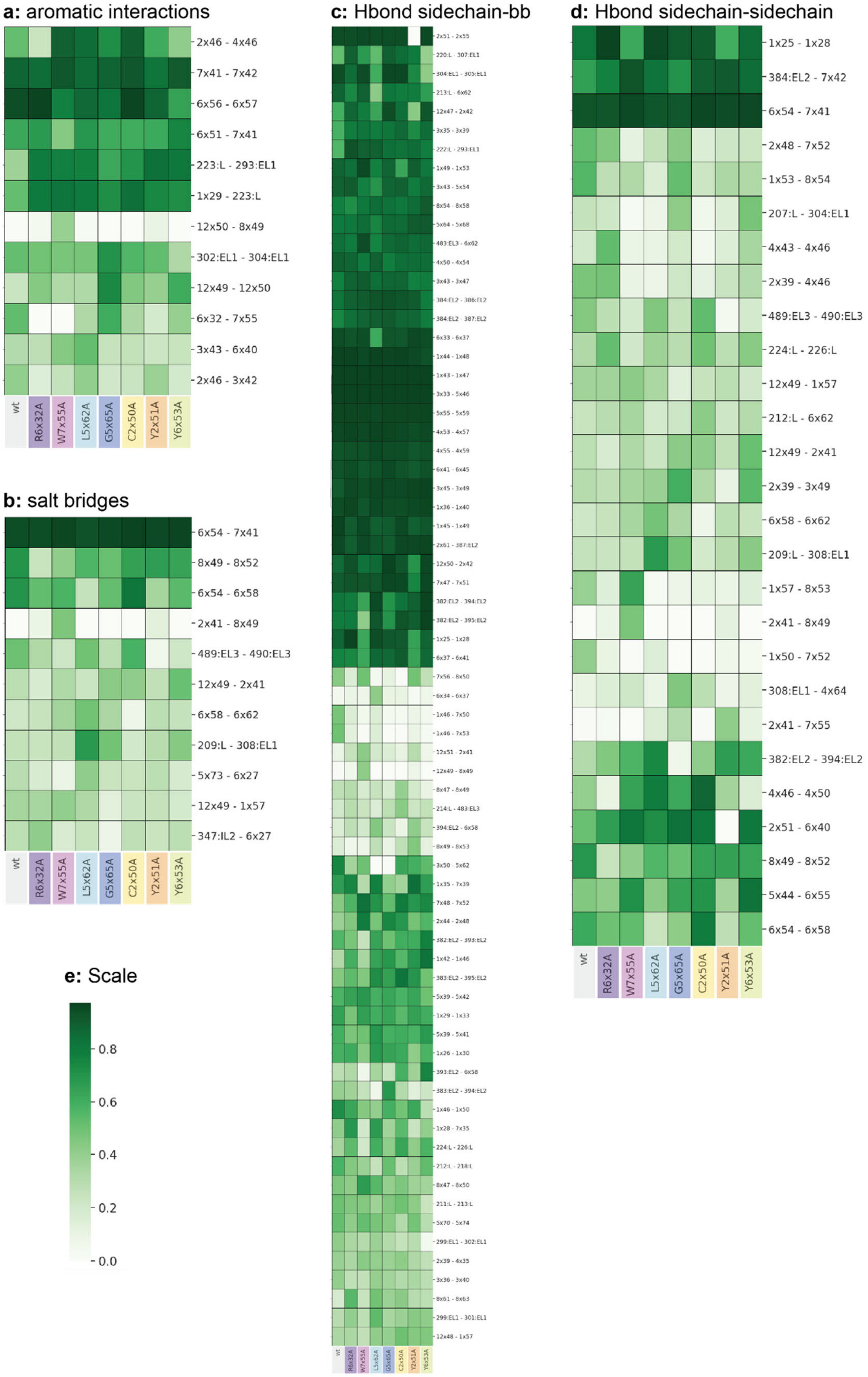

Supplementary Fig. S15: Interaction fingerprint heatmaps from wild-type FZD_5 and micro-switch mutant MD simulations. The interactions were calculated over all three replicas based on geometric orientation of amino acid side chains using getcontacts (https://getcontacts.github.io/). All interactions with a frequency above 40 % for at least one of the simulated systems were included into the fingerprint heatmaps and colored according to their frequency. Residues are numbered according to the numbering scheme or indicated as "*residue number : loop number or linker domain L"*. Mutants are colored according to **Fig. 8**. (**a**) Aromatic interactions: π-stacking, t-stacking and π-cation interactions. (**b**) Salt bridges. (**c**) Hydrogen bonds between sidechain and backbone atom. (**d**) Hydrogen bonds between two sidechain atoms. (**e**) Color scale as used to indicate interaction frequencies in the heatmaps. Based on a visual analysis of the heatmaps, only few cluster-specific interaction patterns could be identified. Changes in interaction patterns for the different mutants compared to wild-type FZD_5 or other mutants are not easily correlated to signaling outcomes. The frequency values of this analysis over all three replicas as well as individual replicas can be found in **Supplementary Data 3** and MD trajectories are deposited in the GPCRMD database for further reference.

Supplementary Figure S16

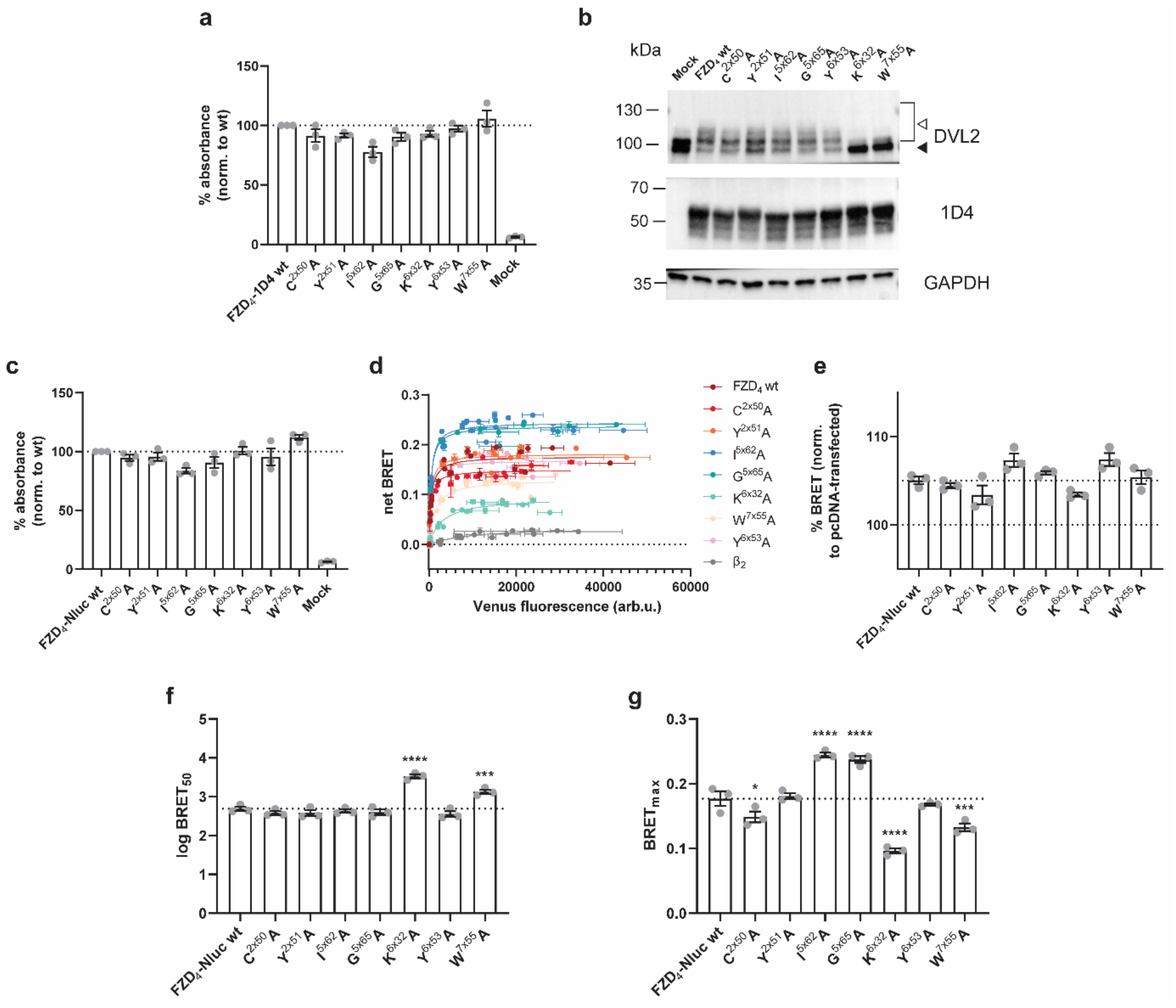

Supplementary Fig. S16: Functional investigation of selected micro-switch mutants in FZD4. Selected state-destabilizing mutations were introduced into the HA-FZD4-1D4 (**a**, **b**) and HA-FZD4-Nluc (**c**-**g**) backbones, respectively, and tested in a panel of constitutive assay paradigms. (**a**, **c**) Assessment of cell surface expression in a whole-cell ELISA targeting the N-terminal HA-tag. Experiments were performed in HEK293A cells, transiently transfected with HA-FZD4-1D4 (**a**, wild-type FZD4 or mutants) or HA-FZD4-Nluc (**c**, wild-type FZD4 or mutants) or pcDNA3.1 (Mock). Data show mean ± SEM of three independent experiments, each performed in triplicate. Data were normalized to the respective FZD4 wt construct for each experiment. Statistical differences between the FZD4 constructs and Mock were assessed using one-way ANOVA (matched, with Geisser-Greenhouse correction), followed by an uncorrected Fisher's LSD post-hoc test. Note that all constructs were significantly expressed at the cell surface. (**b**) Effects of FZD4 micro-switch mutants on the electrophoretic mobility shift of endogenous DVL2. Shown are immunoblots of HEK293A cells transiently transfected with pcDNA3.1 (Mock) or the

indicated HA-FZD_4-1D4 construct. Cell lysates were analyzed for the overexpression-induced electrophoretic mobility shift of DVL2 from basal (filled triangle) to shifted (open triangle) using an anti-DVL2 antibody. Total expression of the transfected FZD_4 constructs was assessed using an anti-1D4 antibody and anti-GAPDH served as a loading control. Immunoblots are representative of two independent experiments. Uncropped blots are shown in the **Source Data File**. (**d**) Recruitment of DEP-Venus to FZD_4-Nluc (wild-type FZD_4 or indicated mutant). Experiments were performed in HEK293A cells, transiently transfected with a constant amount of FZD_4-Nluc (wild-type FZD_4 or mutant) and increasing amounts of DEP-Venus. Data show mean ± SD of three independent experiments (data points from replicates are superimposed), each performed in duplicate. (**e**) % BRET values describing the coupling of G_{12} 4A to FZD_4-Nluc (wild-type FZD_4 or mutant). Data show mean ± SEM of three independent experiments performed in triplicate. For each receptor construct, values obtained in the presence of G_{12} 4A were normalized to a control transfection (same FZD_4-Nluc construct but no G_{12} 4A). Statistical differences between wild-type FZD_4-Nluc and the FZD_4 micro-switch mutants were assessed using one-way ANOVA (matched), followed by Dunnett's post-hoc analysis. (**f**, **g**) log $BRET_{50}$ (**f**) and $BRET_{max}$ (**g**) values extracted from DEP titration experiments (**d**). Data show mean ± SEM of three independent experiments performed in duplicate. Statistical differences between FZD_4-Nluc and the FZD_4 micro-switch mutants were assessed using one-way ANOVA followed by Dunnett's post-hoc analysis. Significance levels are given as follows: * $p < 0.05$, ** $p < 0.01$, *** $p < 0.001$, and **** $p < 0.0001$. Abbreviations: arb. u.: arbitrary units.

Supplementary Figure S17

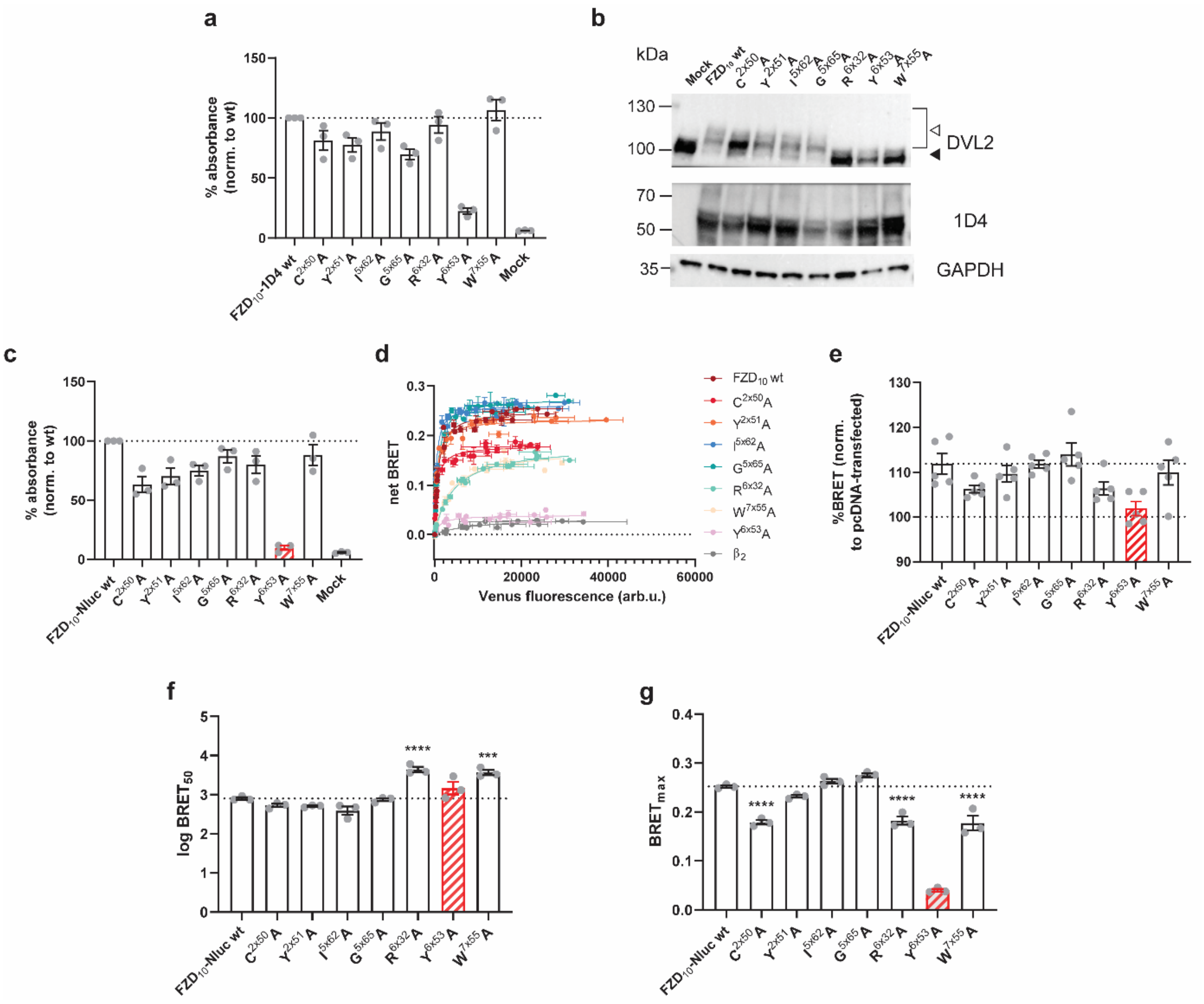

Supplementary Fig. S17: Functional investigation of selected micro-switch mutants in FZD10. Selected state-destabilizing mutations were introduced into the HA-FZD10-1D4 (**a**, **b**) and HA-FZD10-Nluc (**c-g**) backbones, respectively, and tested in a panel of constitutive assay paradigms. (**a**, **c**) Assessment of cell surface expression in a whole-cell ELISA targeting the N-terminal HA-tag. Experiments were performed in HEK293A cells, transiently transfected with HA-FZD10-1D4 (**a**, wild-type FZD10 or mutants) or HA-FZD10-Nluc (**c**, wild-type FZD10 or mutants) or pcDNA3.1 (Mock). Data show mean ± SEM of three independent experiments, each performed in triplicate. Data were normalized to the respective FZD10 wt construct for each experiment. Statistical differences between the FZD10 constructs and Mock were assessed using one-way ANOVA (matched, with Geisser-Greenhouse correction), followed by an uncorrected Fisher's LSD post-hoc test. Note that all constructs except FZD10-Nluc Y^{6x53}A (labeled with a red hatched bar) were significantly expressed at the cell surface. (**b**) Effects of FZD10 micro-switch mutants on the electrophoretic mobility shift of endogenous DVL2. Shown

are immunoblots of HEK293A cells transiently transfected with pcDNA3.1 (Mock) or the indicated HA-FZD_{10}-1D4 construct. Cell lysates were analyzed for the overexpression-induced electrophoretic mobility shift of DVL2 from basal (filled triangle) to shifted (open triangle) using an anti-DVL2 antibody. Total expression of the transfected FZD_{10} constructs was assessed using an anti-1D4 antibody and anti-GAPDH served as a loading control. Immunoblots are representative of two independent experiments. Uncropped blots are shown in the **Source Data File**. (**d**) Recruitment of DEP-Venus to FZD_{10}-Nluc (wild-type FZD_{10} or indicated mutant). Experiments were performed in HEK293A cells, transiently transfected with a constant amount of FZD_{10}-Nluc (wild-type FZD_{10} or mutant) and increasing amounts of DEP-Venus. Data show mean ± SD of three independent experiments (data points from replicates are superimposed), each performed in duplicate. (**e**) % BRET values describing the coupling of G_{13} 4A to FZD_{10}-Nluc (wild-type FZD_{10} or mutant). Data show mean ± SEM of five independent experiments performed in triplicate. For each receptor construct, values obtained in the presence of G_{13} 4A were normalized to a control transfection (same FZD_4-Nluc construct but no G_{13} 4A). Statistical differences between wild-type FZD_{10}-Nluc and the FZD_{10} micro-switch mutants were assessed using one-way ANOVA, followed by Dunnett's post-hoc analysis. FZD_{10}-Nluc $Y^{6x53}A$ was excluded from the statistical analysis due to lack of surface expression (labeled with a red hatched bar). (**f**, **g**) log $BRET_{50}$ (**f**) and $BRET_{max}$ (**g**) values extracted from DEP titration experiments (**d**). Data show mean ± SEM of three independent experiments performed in duplicate. Statistical differences between FZD_4-Nluc and the FZD_4 micro-switch mutants were assessed using one-way ANOVA followed by Dunnett's post-hoc analysis. FZD_{10}-Nluc $Y^{6x53}A$ (labeled with a red hatched bar) was excluded from the statistical analysis due to lack of surface expression. Significance levels are given as follows: * $p < 0.05$, ** $p < 0.01$, *** $p < 0.001$, and **** $p < 0.0001$. Abbreviations: arb. u.: arbitrary units.

Supplementary Figure S18

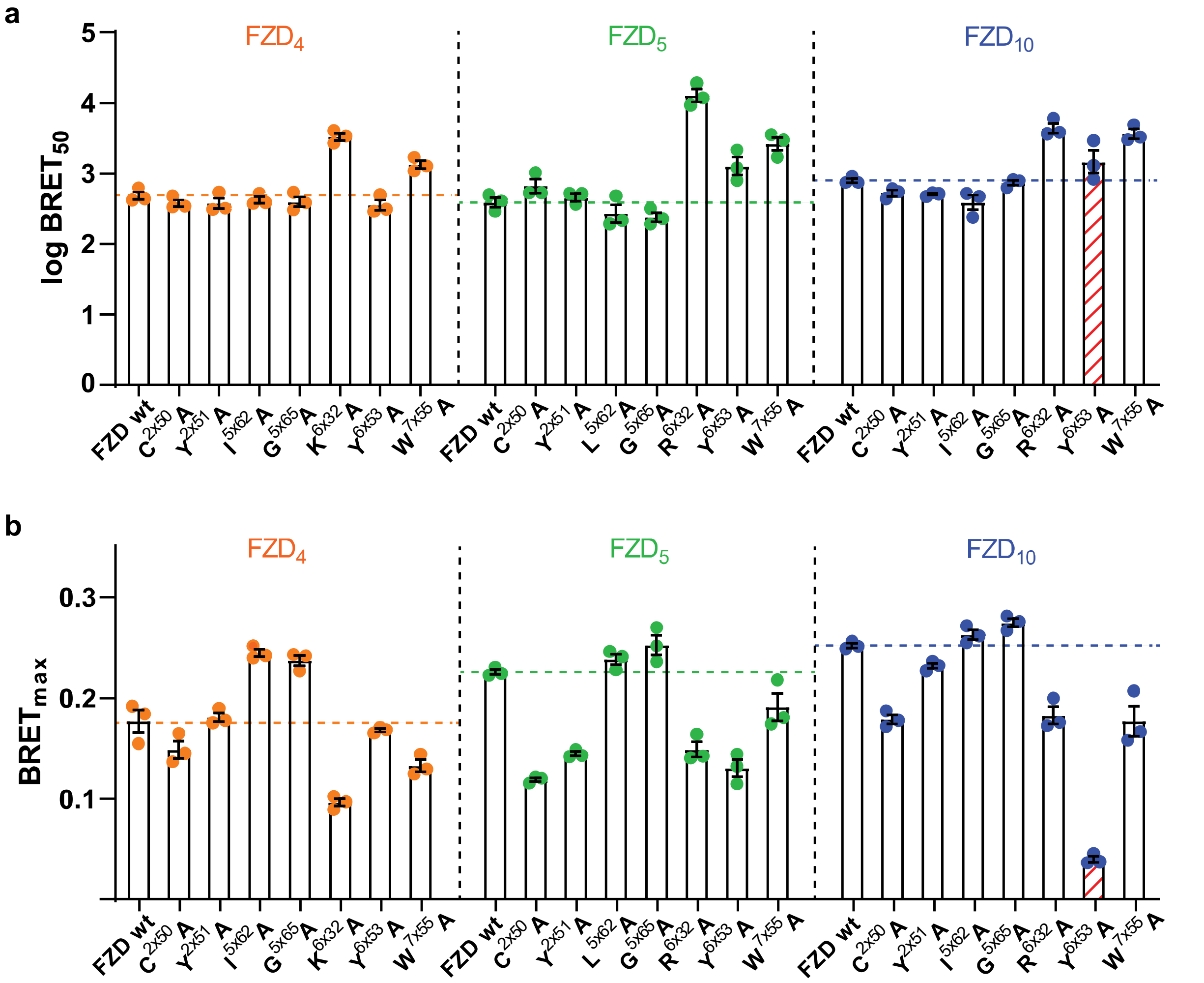

Supplementary Fig. S18: Comparison of parameters from DEP titration experiments between FZD paralogs. Parameters obtained from DEP titrations with FZD_4-Nluc, FZD_5-Nluc, FZD_{10}-Nluc (wild-type FZD and mutants), i.e., log $BRET_{50}$ (**a**) and $BRET_{max}$ (**b**) values, were copied from **Fig. 4g** and **4h** (for FZD_5) and **Supplementary Fig. S16** (for FZD_4) and **S17** (for FZD_{10}), allowing for an easier comparison. Data show mean ± SEM of three independent experiments performed in duplicate, as stated in the respective figure legends. Note: FZD_{10}-Nluc Y^{6x53}A (labeled with a red hatched bar) was not expressed at the cell surface.

Supplementary Table S1

Supplementary Table S1. Primers used in the study.

subst HA FZD_5_for	GCC GGA TTA TGC GGG ATC CGC CTC TAA GGC CC
subst HA FZD_5_rev	ACA TCA TAC GGA TAC CGG CTG CCT TCT CCC GG
FZD_5-*Xba*I_*rev*	GAT CTC TAG ACT CGA GCC GAC GTG GCT CAG AGA CAC
*Xba*I-Nluc_for	GAT CTA TCT AGA TGG AGG TGG CGG TTC TGT CTT CAC ACT CGA AGA TTT CG
Nluc+stop-*Not*I_rev	GAT CGC GGC CGC TTA CGC CAG AAT GCG TTC GCA C
lin FZD_5 K439_for	ACC GAC AAG CTG GAA AAA CTG
lin FZD_5 K439_rev	CTT GGT GCC GCC TTG CTT G
HaloTag FZD_5 K439_for	GTG ATC AAG CAA GGC GGC ACC AAG GAA ATC GGT ACT GGC TTT CC
HaloTag FZD_5 K439_rev	CAT CAG TTT TTC CAG CTT GTC GGT ACC GGA AAT CTC CAG AGT AG
signal seq FZD_for	CTG GCT AGT TAA GCT TCC ACC ATG CGG CTC TGC ATC C
FZD_4-*XbaI*_rev	GAT CTC TAG ACT CGA GCC TAC CAC AGT CTC ACT GCC
FZD_{10}-*XbaI*_rev	GAT CTC TAG ACT CGA GCC CAC GCA GGT GGG CGA C
lin HA-FZD_5-1D4_for	GGT ACC GCC TCC TCG GAT G
lin HA-FZD_5-1D4_rev	CAA TTG ACT CCC TGC AGG AGC
FZD_4 for HA-FZD-1D4_for	ATT CGC TCC TGC AGG GAG TCA ATT GTT CGG GGA CGA GGA AGA G
FZD_4 for HA-FZD-1D4_rev	CTG GCC TCA TCC GAG GAG GCG GTA CCT ACC ACA GTC TCA CTG C
FZD_{10} for HA-FZD-1D4_for	ATT CGC TCC TGC AGG GAG TCA ATT GAT CAG CTC CAT GGA CAT GGA GC
FZD_{10} for HA-FZD-1D4_rev	CTG GCC TCA TCC GAG GAG GCG GTA CCC ACG CAG GTG GGC GAC
FZD_4-Y^{2x51}A_for	TTT CTC AGT ATG TGC GCT AAT ATT TAT AGC AT
FZD_4-Y^{2x51}A_rev	ATG CTA TAA ATA TTA GCG CAC ATA CTG AGA AA
FZD_4-I^{5x62}A_for	ATT GGA ACT TTG TTC GCT GCT GCA GGT TTG GT
FZD_4- I^{5x62}A_rev	ACC AAA CCT GCA GCA GCG AAC AAA GTT CCA AT
FZD_4-G^{5x65}A_for	TGT TCA TTG CTG CAG CTT TGG TGG CCT TGT T
FZD_4-G^{5x65}A_rev	AAC AAG GCC ACC AAA GCT GCA GCA ATG AAC A
FZD_4-K^{6x32}A_for	GAA AGA CTG ATG GTC GCT ATT GGG GTG TTC TCA

FZD_4- $K^{6x32}A$_rev	TGA GAA CAC CCC AAT AGC GAC CAT CAG TCT TTC
FZD_4- $Y^{6x53}A$_for	ATT GCC TGT TAT TTT GCT GAA ATC TCC AAC TG
FZD_4- $Y^{6x53}A$_rev	CAG TTG GAG ATT TCA GCA AAA TAA CAG GCA AT
FZD_4- $W^{7x55}A$_for	ATC ACT TCA GGC ATG GCT ATT TGG TCT GCC AAA
FZD_4- $W^{7x55}A$_rev	TTT GGC AGA CCA AAT AGC CAT GCC TGA AGT GAT
FZD_5-$T^{1x50}A$_for	ATC AGC ACC TCT ACA GCC GTG GCC ACC TTC C
FZD_5-$T^{1x50}A$_rev	GGA AGG TGG CCA CGG CTG TAG AGG TGC TGA T
FZD_5-$T^{1x53}A$_for	TCT ACA ACC GTG GCC GCC TTC CTG ATC GAC A
FZD_5-$T^{1x53}A$_rev	TGT CGA TCA GGA AGG CGG CCA CGG TTG TAG A
FZD_5-$Y^{2x39}A$_for	ATG GAA CGG TTC AGA GCA CCC GAG CGG CCT ATC
FZD_5-$Y^{2x39}A$_rev	GAT AGG CCG CTC GGG TGC TCT GAA CCG TTC CAT
FZD_5-$C^{2x50}A$_for	ATC TTC CTG AGC GCC GCT TAC CTG TGC GTG TC
FZD_5-$C^{2x50}A$_rev	GAC ACG CAC AGG TAA GCG GCG CTC AGG AAG AT
FZD_5-$Y^{2x51}A$_for	TTC CTG AGC GCC TGT GCC CTG TGC GTG TCC CT
FZD_5-$Y^{2x51}A$_rev	AGG GAC ACG CAC AGG GCA CAG GCG CTC AGG AA
FZD_5-$F^{3x29}A$_for	CTG TGC ACC ATC GTG GCT CTG CTG GTG TAC TT
FZD_5-$F^{3x29}A$_rev	AAG TAC ACC AGC AGA GCC ACG ATG GTG CAC AG
FZD_5-$Y^{3x33}A$_for	GTG TTT CTG CTG GTG GCC TTC TTC GGC ATG GC
FZD_5-$Y^{3x33}A$_rev	GCC ATG CCG AAG AAG GCC ACC AGC AGA AAC AC
FZD_5-$M^{3x37}A$_for	GTG TAC TTC TTC GGC GCG GCC AGC TCC ATT TG
FZD_5-$M^{3x37}A$_rev	CAA ATG GAG CTG GCC GCG CCG AAG AAG TAC AC
FZD_5-$W^{3x43}A$_for	GCC AGC TCC ATT TGG GCA GTC ATC CTG AGC CTG
FZD_5-$W^{3x43}A$_rev	CAG GCT CAG GAT GAC TGC CCA AAT GGA GCT GGC
FZD_5-$V^{3x44}A$_for	GCT CCA TTT GGT GGG CCA TCC TGA GCC TGA C
FZD_5-$V^{3x44}A$_rev	GTC AGG CTC AGG ATG GCC CAC CAA ATG GAG C
FZD_5-$W^{3x50}A$_for	ATC CTG AGC CTG ACC GCG TTT CTG GCC GCT GG
FZD_5-$W^{3x50}A$_rev	CCA GCG GCC AGA AAC GCG GTC AGG CTC AGG AT
FZD_5-$L^{3x52}A$_for	AGC CTG ACC TGG TTT GCA GCC GCT GGA ATG AAG

FZD_5-L^{3x52}A_rev	CTT CAT TCC AGC GGC TGC AAA CCA GGT CAG GCT
FZD_5-F^{4x45}A_for	GGC TAC GCC CAG TAT GCT CAT CTG GCC GCC TG
FZD_5-F^{4x45}A_rev	CAG GCG GCC AGA TGA GCA TAC TGG GCG TAG CC
FZD_5-V^{45x52}A_for	CCG GAA TCT GTT ACG CGG GCA ACC AGA ACC T
FZD_5-V^{45x52}A_rev	AGG TTC TGG TTG CCC GCG TAA CAG ATT CCG G
FZD_5-F^{5x46}A_for	AAC AGC CTG AGA GGC GCA GTG CTG GGA CCT CTG
FZD_5-F^{5x46}A_rev	CAG AGG TCC CAG CAC TGC GCC TCT CAG GCT GTT
FZD_5-V^{5x47}A_for	GCC TGA GAG GCT TCG CGC TGG GAC CTC TGG T
FZD_5-V^{5x47}A_rev	ACC AGA GGT CCC AGC GCG AAGC CTC TCA GGC
FZD_5-L^{5x48}A_for	CTG AGA GGC TTC GTG GCA GGA CCT CTG GTG CTG
FZD_5-L^{5x48}A_rev	CAG CAC CAG AGG TCC TGC CAC GAA GCC TCT CAG
FZD_5-P^{5x50}A_for	GGC TTC GTG CTG GGA GCT CTG GTG CTG TAT C
FZD_5-P^{5x50}A_rev	GAT ACA GCA CCA GAG CTC CCA GCA CGA AGC C
FZD_5-G^{5x58}A_for	TGT ATC TGC TCG TGG CCA CCC TGT TTT TGC T
FZD_5-G^{5x58}A_rev	AGC AAA AAC AGG GTG GCC ACG AGC AGA TAC A
FZD_5-L^{5x62}A_for	GTG GGC ACC CTG TTT GCG CTG GCC GGC TTT GT
FZD_5-L^{5x62}A_rev	ACA AAG CCG GCC AGC GCA AAC AGG GTG CCC AC
FZD_5-G^{5x65}A_for	TGT TTT TGC TGG CCG CCT TTG TGT CCC TGT T
FZD_5-G^{5x65}A_rev	AAC AGG GAC ACA AAG GCG GCC AGC AAA AAC A
FZD_5-M^{6x30}A_for	AAG CTG GAA AAA CTG GCG ATC CGC ATC GGC AT
FZD_5-M^{6x30}A_rev	ATG CCG ATG CGG ATC GCC AGT TTT TCC AGC TT
FZD_5-R^{6x32}A_for	GAA AAA CTG ATG ATC GCC ATC GGC ATC TTC AC
FZD_5-R^{6x32}A_rev	GTG AAG ATG CCG ATG GCG ATC ATC AGT TTT TC
FZD_5-G^{6x34}A_for	TGA TGA TCC GCA TCG CCA TCT TCA CCC TGC T
FZD_5-G^{6x34}A_rev	AGC AGG GTG AAG ATG GCG ATG CGG ATC ATC A
FZD_5-Y^{6x51}A_for	ATC GTG GTG GCT TGC GCC CTG TAC GAA CAG CA
FZD_5-Y^{6x51}A_rev	TGC TGT TCG TAC AGG GCG CAA GCC ACC ACG AT
FZD_5-L^{6x52}A_for	GTG GTG GCT TGC TAC GCG TAC GAA CAG CAC TA

FZD_5-L^{6x52}A_rev	TAG TGC TGT TCG TAC GCG TAG CAA GCC ACC AC
FZD_5-Y^{6x53}A_for	GTG GCT TGC TAC CTG GCA GAA CAG CAC TAC AGA
FZD_5-Y^{6x53}A_rev	TCT GTA GTG CTG TTC TGC CAG GTA GCA AGC CAC
FZD_5-W^{6x61}A_for	CAC TAC AGA GAG AGC GCA GAA GCC GCT CTG ACA
FZD_5-W^{6x61}A_rev	TGT CAG AGC GGC TTC TGC GCT CTC TCT GTA GTG
FZD_5-K^{7x41}A_for	TGG GTC CTG ATG CTG GCG TAC TTT ATG TGC CT
FZD_5-K^{7x41}A_rev	AGG CAC ATA AAG TAC GCC AGC ATC AGG ACC CA
FZD_5-M^{7x44}A_for	ATG CTG AAG TAC TTT GCG TGC CTG GTC GTG GG
FZD_5-M^{7x44}A_rev	CCC ACG ACC AGG CAC GCA AAG TAC TTC AGC AT
FZD_5-W^{7x55}A_for	ATC ACC AGC GGC GTT GCG ATT TGG AGC GGC AA
FZD_5-W^{7x55}A_rev	TTG CCG CTC CAA ATC GCA ACG CCG CTG GTG AT
FZD_5-W^{8x54}A_for	AAG ACC GTG GAA AGC GCG CGG AGG TTC ACC AG
FZD_5-W^{8x54}A_rev	CTG GTG AAC CTC CGC GCG CTT TCC ACG GTC TT
FZD_{10}-C^{2x50}A_for	ATC TTC CTC TCC ATG GCT TAC TGC GTC TAC TCC
FZD_{10}-C^{2x50}A_rev	GGA GTA GAC GCA GTA AGC CAT GGA GAG GAA GAT
FZD_{10}-Y^{2x51}A_for	TTC CTC TCC ATG TGC GCC TGC GTC TAC TCC GT
FZD_{10}-Y^{2x51}A_rev	ACG GAG TAG ACG CAG GCG CAC ATG GAG AGG AA
FZD_{10}-I^{5x62}A_for	ATC GGC ACG TCC TTC GCC CTC TCG GGC TTC GT
FZD_{10}- I^{5x62}A_rev	ACG AAG CCC GAG AGG GCG AAG GAC GTG CCG AT
FZD_{10}-G^{5x65}A_for	CCT TCA TCC TCT CGG CTT TCG TGG CCC TGT TC
FZD_{10}-G^{5x65}A_rev	GAA CAG GGC CAC GAA AGC CGA GAG GAT GAA GG
FZD_{10}-R^{6x32}A_for	GAG AAG CTC ATG GTG GCT ATC GGG CTC TTC TC
FZD_{10}- R^{6x32}A_rev	GAG AAG AGC CCG ATA GCC ACC ATG AGC TTC TC
FZD_{10}- Y^{6x53}A_for	ATC GCC TGC TAC TTT GCC GAA CGC CTC AAC AT
FZD_{10}- Y^{6x53}A_rev	ATG TTG AGG CGT TCG GCA AAG TAG CAG GCG AT
FZD_{10}- W^{7x55}A_Q5_for	CAG CGG GAT GGC TAT TTG GAC CTC CAA G
FZD_{10}- W^{7x55}A_Q5_rev	GTG ATC CCC ACC ACC AGC

Supplementary Table S2

Supplementary Table S2. p values for indicated FZD_5 micro-switch mutants from the different ELISA assays (results from **Fig. 1C**, **Supplementary Fig. S3** and **Supplementary Fig. S5C**). Statistical differences to pcDNA3.1-transfected HEK293A cells were tested using one-way ANOVA (matched, with Geisser-Greenhouse correction) followed by uncorrected Fisher's LSD post-hoc test as described in the figure legends. Mutants marked in red were found not to be significantly expressed compared to pcDNA3.1-transfected cells. ns, not significant; *: p < 0.05; **: p < 0.01; ***: p < 0.001; ****: p < 0.0001.

	*HA-FZD₅ micro-switch surface expression (**Fig. 1C**); N = 5*	*HA-FZD₅-Nluc micro-switch surface expression (**Suppl. Fig. S3**); N = 4*	*HA-FZD₅-Halo-Nluc micro-switch surface expression (**Suppl. Fig. S5C**); N = 4*
$T^{1x50}A$	< 0.0001; ****	0.0003; ***	0.0004; ***
$T^{1x53}A$	< 0.0001; ****	0.0013; **	0.0024; **
$Y^{2x39}A$	< 0.0001; ****	0.0023; **	0.0111; *
$C^{2x50}A$	0.0034; **	0.0151; *	0.0177; *
$Y^{2x51}A$	0.0022; **	0.0408; *	0.0096; **
$F^{3x29}A$	< 0.0001; ****	0.0087; **	0.0017; **
$Y^{3x33}A$	0.9326; ns	-	-
$M^{3x37}A$	< 0.0001; ****	0.0097; **	0.0101; *
$W^{3x43}A$	< 0.0001; ****	0.0038; **	0.0012; **
$V^{3x44}A$	< 0.0001; ****	0.0260; *	0.0064; **
$W^{3x50}A$	0.7890; ns	-	-
$L^{3x52}A$	0.0004; ***	0.0098; **	0.0068; **
$F^{4x45}A$	0.0298; *	0.0203; *	0.0051; **
$V^{45x52}A$	< 0.0001; ****	0.0021; **	0.0049; **
$F^{5x46}A$	0.6189; ns	-	-
$V^{5x47}A$	0.1951; ns	-	-
$L^{5x48}A$	0.0005; ***	0.0104; *	0.0055; **
$P^{5x50}A$	0.9166; ns	-	-
$G^{5x58}A$	0.0070; **	0.0014; **	0.0025; **
$L^{5x62}A$	< 0.0001; ****	0.0016; **	0.0002; ***
$G^{5x65}A$	< 0.0001; ****	0.0019; **	0.0020; **
$M^{6x30}A$	<0.0001; ****	0.0002; ***	< 0.0001; ****
$R^{6x32}A$	< 0.0001; ****	0.0008; ***	0.0021; **
$G^{6x34}A$	< 0.0001; ****	0.0002; ***	0.0057; **
$Y^{6x51}A$	< 0.0001; ****	0.0031; **	0.0026; **
$L^{6x52}A$	< 0.0001; ****	0.0002; ***	< 0.0001; ****
$Y^{6x53}A$	0.0204; *	0.0242; *	0.0176; *
$W^{6x61}A$	0.0008; ***	0.0049; **	0.0299; *
$K^{7x41}A$	0.9056; ns	-	-
$M^{7x44}A$	< 0.0001; ****	0.0148; *	0.0014; **
$W^{7x55}A$	< 0.0001; ****	< 0.0001; ****	0.0005; ***
$W^{8x54}A$	< 0.0001; ****	0.0009; ***	0.0033; ***

Supplementary Table S3

Supplementary Table S3. log $BRET_{50}$ and $BRET_{max}$ values from DEP-Venus titration experiments to wild-type FZD_5-Nluc (wt) or FZD_5-Nluc micro-switch mutants (**Fig. 4**). Data represent mean ± SEM of three independent experiments performed in duplicate.

	*log $BRET_{50}$ ± SEM (**Fig. 4G**), N = 3*	*$BRET_{max}$ ± SEM (**Fig. 4H**), N = 3*
FZD_5 wt	2.59 ± 0.07	0.226 ± 0.002
$T^{1x50}A$	3.22 ± 0.12	0.216 ± 0.003
$T^{1x53}A$	3.13 ± 0.12	0.187 ± 0.006
$Y^{2x39}A$	3.39 ± 0.08	0.175 ± 0.011
$C^{2x50}A$	2.82 ± 0.10	0.119 ± 0.002
$Y^{2x51}A$	2.67 ± 0.05	0.145 ± 0.002
$F^{3x29}A$	2.51 ± 0.03	0.191 ± 0.001
$M^{3x37}A$	3.03 ± 0.10	0.163 ± 0.006
$W^{3x43}A$	2.61 ± 0.04	0.218 ± 0.008
$V^{3x44}A$	2.49 ± 0.06	0.204 ± 0.006
$L^{3x52}A$	2.83 ± 0.07	0.177 ± 0.005
$F^{4x45}A$	3.07 ± 0.11	0.138 ± 0.007
$V^{45x52}A$	2.53 ± 0.15	0.191 ± 0.006
$L^{5x48}A$	2.72 ± 0.04	0.158 ± 0.005
$G^{5x58}A$	2.55 ± 0.11	0.212 ± 0.007
$L^{5x62}A$	2.44 ± 0.13	0.238 ± 0.005
$G^{5x65}A$	2.39 ± 0.07	0.253 ± 0.010
$M^{6x30}A$	3.20 ± 0.08	0.209 ± 0.005
$R^{6x32}A$	4.11 ± 0.09	0.149 ± 0.008
$G^{6x34}A$	2.43 ± 0.16	0.227 ± 0.008
$Y^{6x51}A$	2.51 ± 0.19	0.197 ± 0.011
$L^{6x52}A$	2.72 ± 0.01	0.225 ± 0.009
$Y^{6x53}A$	3.11 ± 0.13	0.131 ± 0.009
$W^{6x61}A$	2.72 ± 0.07	0.143 ± 0.007
$M^{7x44}A$	2.19 ± 0.10	0.218 ± 0.001
$W^{7x55}A$	3.42 ± 0.09	0.191 ± 0.014
$W^{8x54}A$	2.93 ± 0.04	0.188 ± 0.010

Supplementary Table S4

Supplementary Table S4. % BRET values from experiments with G_q 4A, Venus-βγ and wild-type FZD_5-Nluc (wt) or FZD_5-Nluc micro-switch mutants (**Fig. 5**). For each experiment, raw BRET ratios were normalized to the BRET ratios from cells transfected with the respective FZD_5-Nluc mutant but no G_q 4A. Data represent mean ± SEM of three independent experiments performed in triplicate.

	*% BRET (norm. to Mock) ± SEM (**Fig. 5**), N = 3*
FZD_5 wt	110.7 ± 0.5
$T^{1x50}A$	111.0 ± 1.2
$T^{1x53}A$	105.9 ± 0.8
$Y^{2x39}A$	108.8 ± 0.6
$C^{2x50}A$	101.4 ± 0.6
$Y^{2x51}A$	101.1 ± 1.1
$F^{3x29}A$	108.2 ± 0.6
$M^{3x37}A$	103.2 ± 0.6
$W^{3x43}A$	108.9 ± 1.6
$V^{3x44}A$	106.9 ± 1.4
$L^{3x52}A$	103.4 ± 0.6
$F^{4x45}A$	102.2 ± 0.3
$V^{45x52}A$	104.9 ± 0.5
$L^{5x48}A$	102.4 ± 0.7
$G^{5x58}A$	106.2 ± 0.6
$L^{5x62}A$	113.3 ± 0.7
$G^{5x65}A$	115.6 ± 2.1
$M^{6x30}A$	111.6 ± 1.3
$R^{6x32}A$	106.5 ± 0.2
$G^{6x34}A$	108.9 ± 1.0
$Y^{6x51}A$	106.3 ± 0.6
$L^{6x52}A$	109.3 ± 0.9
$Y^{6x53}A$	100.4 ± 1.3
$W^{6x61}A$	102.8 ± 0.7
$M^{7x44}A$	107.8 ± 0.9
$W^{7x55}A$	111.0 ± 0.5
$W^{8x54}A$	105.1 ± 0.6

Supplementary Table S5

Supplementary Table S5. $BRET_0$ values (± SD of linear fit) from experiments with the FZD_5-Halo-Nluc conformational sensor (wild-type (wt) and micro-switch mutants, see **Fig. 6** and **Supplementary Fig. S6**), R^2 of linear fits and p values of runs test for linear correlation to test for deviation from linearity ($p < 0.05$ was considered significant) in **Supplementary Fig. S6**.

	*$BRET_0$ ± SD (**Fig. 6**), N = 5*	*R^2 of linear fit*	*p values of runs test for deviation from linear correlation*
FZD_5 wt	0.051 ± 0.002	0.6036	0.5136
$T^{1x50}A$	0.056 ± 0.002	0.6906	0.5136
$T^{1x53}A$	0.069 ± 0.003	0.7244	0.2086
$Y^{2x39}A$	0.056 ± 0.002	0.6111	0.1492
$C^{2x50}A$	0.080 ± 0.002	0.7789	0.0513
$Y^{2x51}A$	0.070 ± 0.003	0.5074	0.7622
$F^{3x29}A$	0.056 ± 0.002	0.8021	0.5136
$M^{3x37}A$	0.068 ± 0.002	0.7979	0.1492
$W^{3x43}A$	0.062 ± 0.002	0.8518	0.1492
$V^{3x44}A$	0.063 ± 0.001	0.8883	0.1492
$L^{3x52}A$	0.073 ± 0.002	0.7341	0.4545
$F^{4x45}A$	0.075 ± 0.003	0.7460	0.5664
$V^{45x52}A$	0.061 ± 0.001	0.8338	0.1492
$L^{5x48}A$	0.075 ± 0.002	0.7535	0.1492
$G^{5x58}A$	0.063 ± 0.002	0.7962	0.1492
$L^{5x62}A$	0.053 ± 0.002	0.6447	0.1492
$G^{5x65}A$	0.058 ± 0.002	0.7222	0.1492
$M^{6x30}A$	0.054 ± 0.002	0.6317	0.3427
$R^{6x32}A$	0.056 ± 0.002	0.6725	0.5664
$G^{6x34}A$	0.056 ± 0.001	0.8382	0.1492
$Y^{6x51}A$	0.062 ± 0.001	0.8702	0.5136
$L^{6x52}A$	0.053 ± 0.001	0.8168	0.7622
$Y^{6x53}A$	0.106 ± 0.003	0.8446	0.1492
$W^{6x61}A$	0.077 ± 0.002	0.8463	0.3427
$M^{7x44}A$	0.050 ± 0.002	0.7337	0.2960
$W^{7x55}A$	0.054 ± 0.002	0.6142	0.1492
$W^{8x54}A$	0.065 ± 0.003	0.6611	0.5664

Supplementary Table S6

Supplementary Table S6: Templates used for the modeling of the FZD_5 structure. Specific regions were excluded or included as indicated due to lower resolution or improved model quality.

Template PDB ID	Protein	Comments
6WW2 [1]	FZD_5	R193, L417-I448, A480-C486 not aligned
4N4W [2]	SMO	G353-F360 not aligned
4JKV [3]	SMO	G353-F360 not aligned
6BD4 [4]	FZD_4	C181-L202, M342-F347, A407-M434, S460-M474, I489-M493 not aligned
AlphaFold model [5,6]	$hFZD_5$	Only G180-S226, E479-W501

Supplementary References

1 Tsutsumi, N., Mukherjee, S., Waghray, D., et al. Structure of human Frizzled5 by fiducial-assisted cryo-EM supports a heterodimeric mechanism of canonical Wnt signaling. *eLife* 9:e58464 (2020). https://doi.org/10.7554/eLife.58464

2 Wang, C., Wu, H., Evron, T. et al. Structural basis for Smoothened receptor modulation and chemoresistance to anticancer drugs. *Nat Commun* 5, 4355 (2014). https://doi.org/10.1038/ncomms5355

3 Wang, C., Wu, H., Katritch, V. et al. Structure of the human smoothened receptor bound to an antitumour agent. *Nature* 497, 338–343 (2013). https://doi.org/10.1038/nature12167

4 Yang, S., Wu, Y., Xu, TH. et al. Crystal structure of the Frizzled 4 receptor in a ligand-free state. *Nature* 560, 666–670 (2018). https://doi.org/10.1038/s41586-018-0447-x

5 Jumper, J., Evans, R., Pritzel, A. et al. Highly accurate protein structure prediction with AlphaFold. *Nature* 596, 583–589 (2021). https://doi.org/10.1038/s41586-021-03819-2

6 Varadi, M., Anyango, S., Deshpande, M., et al., AlphaFold Protein Structure Database: massively expanding the structural coverage of protein-sequence space with high-accuracy models. *Nucleic Acids Res*, 50, D1, D439–D444 (2022). https://doi.org/10.1093/nar/gkab1061
