# Supplementary material for: Pathway selectivity in Frizzleds is achieved by conserved micro-switches defining pathway-determining, active conformations
Source: Nat Commun. 2023 Jul 29;14:4573. doi: 10.1038/s41467-023-40213-0 (PMC10387068; doi:10.1038/s41467-023-40213-0)
Supplement: Supplementary file 3 — Description of Additional Supplementary Files [file 41467_2023_40213_MOESM3_ESM.pdf]

File Name: Supplementary Data 1

Description: Prediction of state-stabilizing residues in Class F GPCRs using the two analysis tools (“state affecting design tool” and “structure comparison tool”) provided by the GPCRdb.

File Name: Supplementary Data 2

Description: Raw data for Supplementary Fig. S7 presenting the combined effects of receptor mutations in FZD5 on the assessed experimental parameters.

File Name: Supplementary Data 3

Description: Frequency values of the analysis on the interaction fingerprint heatmaps from wild-type FZD5 and micro-switch mutant MD simulations shown in Supplementary Fig. S15.

File Name: Supplementary Data 4

Description: A list of all primers used in this study.
